# Hyaluronic acid-modified liposomes Potentiated *in-vivo* anti-hepatocellular carcinoma of icaritin

**DOI:** 10.3389/fphar.2024.1437515

**Published:** 2024-07-11

**Authors:** Xiaoduan Sun, Zhenzhen He, Ruilin Lu, Zhongbing Liu, Sawitree Chiampanichayakul, Songyot Anuchapreeda, Jun Jiang, Singkome Tima, Zhirong Zhong

**Affiliations:** ^1^ Department of Medical Technology, Faculty of Associated Medical Sciences, Chiang Mai University, Chiang Mai, Thailand; ^2^ Department of Pharmacy, The Affiliated Hospital of Southwest Medical University, Luzhou, China; ^3^ Key Laboratory of Medical Electrophysiology, Ministry of Education, School of Pharmacy, Southwest Medical University, Luzhou, China; ^4^ Suining First People’s Hospital, Suining, China; ^5^ Cancer Research Unit of Associated Medical Sciences (AMS-CRU), Chiang Mai University, Chiang Mai, Thailand; ^6^ Center of Excellence in Pharmaceutical Nanotechnology, Faculty of Pharmacy, Chiang Mai University, Chiang Mai, Thailand; ^7^ Department of General Surgery (Thyroid Surgery), The Affiliated Hospital of Southwest Medical University, Luzhou, China; ^8^ Central Nervous System Drug Key Laboratory of Sichuan Province, Luzhou, China

**Keywords:** hepatocellular carcinoma, liposomes, icaritin, hyaluronic acid, CD44

## Abstract

**Introduction:** Icaritin (ICT), a promising anti-hepatocellular carcinoma (HCC) prenylated flavonoid, is hindered from being applied due to its low water solubility and high lipophilicity in poorly differentiated HCC which is associated with upregulation of CD44 isoforms. Thus, hyaluronic acid (HA), a natural polysaccharide with high binding ability to CD44 receptors, was used to formulate a modified liposome as a novel targeted ICT-delivery system for HCC treatment.

**Methods:** The ICT-Liposomes (Lip-ICT) with and without HA were prepared by a combined method of thin-film dispersion and post-insertion. The particle size, polydispersity (PDI), zeta potential, encapsulation efficacy (%EE), drug loading content (%DLC), and *in vitro* drug release profiles were investigated for physicochemical properties, whereas MTT assay was used to assess cytotoxic effects on HCC cells, HepG2, and Huh7 cells. Tumor bearing nude mice were used to evaluate the inhibitory effect of HA-Lip-ICT and Lip-ICT *in vivo*.

**Results:** Lip-ICT and HA-Lip-ICT had an average particle size of 171.2 ± 1.2 nm and 208.0 ± 3.2 nm, with a zeta potential of −13.9 ± 0.83 and −24.8 ± 0.36, respectively. The PDI resulted from Lip-ICT and HA-Lip-ICT was 0.28 ± 0.02 and 0.26 ± 0.02, respectively. HA-Lip-ICT demonstrated higher *in vitro* drug release when pH was dropped from 7.4 to 5.5, The 12-h release rate of ICT from liposomes increased from 30% at pH7.4 to more than 60% at pH5.5. HA-Lip-ICT displayed higher toxicity than Lip-ICT in both HCC cells, especially Huh7with an IC^50^ of 34.15 ± 2.11 μM. The *in vivo* tissue distribution and anti-tumor experiments carried on tumor bearing nude mice indicated that HA-Lip- ICT exhibited higher tumor accumulation and achieved a tumor growth inhibition rate of 63.4%.

**Discussion:** The nano-sized Lip-ICT was able to prolong the drug release time and showed long-term killing HCC cells ability. Following conjugation with HA, HA-Lip-ICT exhibited higher cytotoxicity, stronger tumor targeting, and tumor suppression abilities than Lip-ICT attributed to HA-CD44 ligand-receptor interaction, increasing the potential of ICT to treat HCC.

## Graphical Abstract

**Figure:**
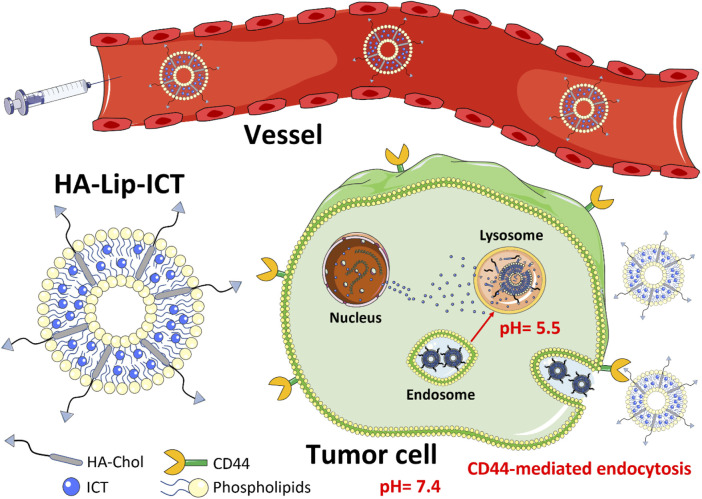
Schematic overview of the hyaluronic acid-modified liposomes loaded with icaritin (HA-Lip-ICT) and its proposed anti-tumor mechanism.

## 1 Introduction

Liver cancer stands as the 6th most prevalent malignancy globally, ranking second among cancer related fatalities. Hepatocellular carcinoma (HCC), responsible for 80% of primary liver cancer cases, which has a bad prognosis with less than 12% of patients having a 5-year overall survival rate (Chen et al., 2016). Nowadays, the foremost methods in treating HCC are surgery and transplantation. Nonetheless, the majority of HCC cases reach inoperable advanced stages where surgical intervention becomes unfeasible, particularly in regions like China (Torre et al., 2015; Chen et al., 2016). Moreover, following surgical resection, the outlook for HCC in the long term remains severe, and the persistent risk of cancer relapse or metastasis poses a substantial hurdle (El-Serag and Rudolph, 2007). Conventional chemotherapy drugs, like cisplatin and its counterparts, consistently yield poor responses in advanced-stage HCC. Moreover, acquired chemotherapy resistance and side effects hinder the long-term use of these therapeutic compounds (Singh et al., 2014). Hence, there’s urgent need to find new anti-cancer agents comes from natural plants (Cragg and Newman, 2005). Natural compounds are very important sources for exploration of anti-cancer drugs (Newman, 2008).

Icaritin (ICT), a prenylated flavonoid, comes from plants named Epimedium genus within the Berberidaceae family. Herba Epimedii extracts are widely applied in traditional Chinese medicine due to their tonic and aphrodisiac characteristics (Sze et al., 2010). ICT is presently in the phase three clinical trial stage for treating advanced HCC, supported by a robust foundation of preclinical and clinical evidence (Bailly, 2020). The drug’s anti-tumor efficacy originates from its ability to influence multiple signaling factors within cancer cells, primarily the chemokine receptor CXCR4, NF-κB and transcription factors STAT3, as well as the estrogen receptor splice variant ERα36 (Tiong et al., 2012). Recent research has associated additional factors, such as various microRNAs, the production of ROS, and the regulation of sphingosine kinase-1 (Lu et al., 2017). Furthermore, ICT interacts with the RAGE-HMGB1 pathway and manages the crosstalk between apoptosis and autophagy, thereby enhancing its anti-cancer capabilities (Liu et al., 2019). Additionally, ICT triggers substantial changes in the tumor’s surroundings, fostering an immune response (Hao et al., 2019). Taken together, these diverse biochemical and cellular features provide a strong activity profile for ICT, making it a valuable option for treating HCC. However, the clinical application of ICT for cancer treatment is hindered by its low water solubility and limited bioavailability. It is crucial to develop new strategies aimed at increasing aqueous solubility and enhancing the bioavailability of ICT (Chang et al., 2012; Y; Li et al., 2013).

Nanotechnology has facilitated the enhancement of the efficacy of drugs in liver cancer treatment. Using nanocarriers as drug delivery systems is a popular strategy for delivering hydrophobic drugs to specific tissues more effectively and enhancing the dissolution of drugs (Sabit et al., 2022). This can be achieved through the utilization of diverse nanocarrier-mediated drug delivery systems, which subsequently enable reduced drug dosages to achieve higher therapeutic effects, minimize systemic toxicity risks, prolong drug release over days following a single administration, and improve specific targeting to cancer cells (Mitchell et al., 2021). Among these, liposomes stand out as the most promising for clinical applications. Generally, liposomes were used to enhance drug dissolution by enclosing poorly soluble drugs within the hydrophobic bilayer (Lee, 2020). Furthermore, liposomes demonstrated favorable biodegradability and biocompatibility, thus avoiding harm to healthy cells (Cheng et al., 2020).

Modified liposomes have been introduced with various specific ligands to enable additional functions. Hyaluronic acid (HA) holds significant potential as a natural material for liposomal functionalization due to its strong affinity to CD44 receptors, which show increased expression in cancerous cells (Akim et al., 2021; Antonio and Trídico, 2021). As a result of this affinity, liposomes can be delivered to cancer cells via receptor-ligand interactions (Kesharwani et al., 2021). HA has found extensive application in formulating dendrimers, micelles and liposomes because of its remarkable hydrophilicity and biocompatibility (Patra et al., 2018; Majumder et al., 2019; Su and Peter, 2020). As a result, utilizing a hydrophilic HA layer on liposomes provides them with physiological stability and imparts active targeting potential to liposomal nanocarriers, facilitating their internalization into cancer cells through HA/CD44 interactions.

It has been reported that the upregulation of CD44 correlates with poorly differentiated HCC and reduced survival rates (Endo and Terada, 2000). CD44 is a cell surface receptor known to have higher expression levels in several solid tumors than in normal tissues. Previous studies indicated the significance of CD44 in sustaining cancer stem cells (CSCs) and its role in governing oxidative stress levels in human HCC cell lines, such as Huh7 (Asai et al., 2019). Utilizing the specific interaction between CD44 and HA, a targeted drug delivery system can be developed for treating HCC.

Hence, our primary objective was to create a cholesterol-tri(ethyleneglycol)-hyaluronic acid conjugate (HA-Chol) to fabricate hyaluronic acid-decorated liposomes (HA-Lip) for the purpose of precisely delivering ICT. Cholesterol was chosen as the molecule for conjugation with HA due to its prominent role for the liposome fluidity and there’s a free secondary hydroxyl group in its structure, it offers versatility for chemical alterations (Ohvo-Rekilä et al., 2002). We hypothesized that hyaluronic acid-coated liposomes containing ICT (HA-Lip-ICT) could effectively avoid the low water solubility and low bioavailability of ICT. Moreover, it would enhance *in vivo* tumor targeting and demonstrate remarkable anti-tumor efficacy against liver cancer through CD44-mediated endocytosis. In this study, the characteristics of HA-Lip-ICT were explored and its anti-tumor efficacy on multiple HCC cell models was assessed, including cytotoxicity, cell cycle arrest and apoptosis. Consequently, the intrinsic molecular mechanism was examined by which HA-Lip-ICT triggers cell cycle arrest and initiates apoptosis using Western blotting. Finally, *in vivo* biodistribution analysis, preliminary safety evaluations and tumor growth inhibition assessments were conducted using BALB/C nude mice bearing Huh7 tumor models.

## 2 Materials and methods

### 2.1 Materials

Icaritin was purchased from Plant Origin Biological Co., Ltd. (Nanjing, China; purity = 98%). Standard icaritin was offered by Chengdu Purechem-Standard Biotech Co., Ltd. (Chengdu, China; purity ≥98%). Cholesterol was given by Solarbio Co. (Beijing, China). Hyaluronic acid (HA, [0.5–1.5] × 10^6^ Da) was obtained from Shanghai Macklin Biochemical Technology Co., Ltd. (Shanghai, China). The Annexin V-FITC/PI apoptosis detection kit was purchased from Solarbio Life Sciences (Beijing, China). Fetal bovine serum (FBS) and trypsin were obtained from Gibco-BRL (New York, United States). Additionally, 3-(4,5-dimethylthiazol-2-yl)-2,5-diphenyltetrazolium bromide (MTT), 1,1-dioctadecyl-3,3,3,3-tetramethylindotricarbocyanine iodide (DiR) and 4′,6-diamidino-2-phenylin-dole dihydrochloride (DAPI) were obtained from Biyuntian Biotechnology (Shanghai, China). Coumarin-6 was acquired from Ruixi Biotechnology (Xi’an, China). Rabbit polyclonal anti-GAPDH, goat anti-mouse immunoglobulin G (IgG), goat anti-rabbit IgG, as well as rabbit polyclonal antibodies against CD44 were obtained from ABclonal Technology (Wuhan, China). The hyaluronic acid-modified cholesterol (HA-Chol) synthesis routes are shown in Supplementary Figure S1 and the details are listed in it too.

### 2.2 Cell culture

The HCC cell lines of human origin (Huh7 and HepG2), along with the normal human liver cell line (L02), were procured from The Chinese Academy of Sciences’ Cell Bank in Shanghai, China, and cultured in DMEM medium containing 10% FBS, 100 IU/mL penicillin and 100 μg/mL streptomycin at 37°C with the condition of 5% CO_2_.

### 2.3 Formulation and characterization of liposomes encapsulating ICT

Liposomes were formulated through a combined method involving mechanical extrusion and thin-film dispersion techniques, using key ingredients, such as soybean lecithin, cholesterol and HA-Chol. Lipids and ICT were dissolved in 10 mL chloroform and methanol (1:1 v/v) then the mixture was transferred to a round-bottom flask. Organic solvent was eliminated with vacuum rotary evaporator till a thin film formed. The thin film was additionally subjected to vacuum drying overnight to ensure there’s no organic solvent. Then, phosphate-buffered solution (PBS, pH 7.4) was used to rehydrate the dried thin film. Lastly, the liposomal suspensions were extruded through the Avanti^®^ Mini-Extruder with a 200-nm polycarbonate membrane to get the desired size-distribution liposomes. The empty liposomes (empty Lip) without ICT were prepared similarly without the addition of ICT. HA-Lip-ICT was formulated using post-insertion method wherein the HA-Chol conjugate was embedded into the lipid bilayer (L. Cheng et al., 2014). An exact amount of HA-Chol conjugate (10% of the total lipid content) was incubated with the liposomes mentioned above for 30 min at 50°C.

Liposome morphology was observed using transmission electron microscopy (TEM, Hitachi HT-7700, Japan). Particle size and the zeta potential of liposomes were assessed by DLS (Zetasizer Nano ZS90, Malvern Instruments, Malvern, United Kingdom). Fourier transform infrared (FTIR) spectra were obtained using an IRAffinity-1S system (Shimadzu Technology, Kyoto, Japan) at frequencies of 500–4000 cm^–1^.

Encapsulation efficiency (EE) and drug loading content (DLC) were determined using ultrafiltration tubes (MWCO = 3 kDa). Unencapsulated ICT (W_free_) was measured using HPLC. The mobile phase were water and acetonitrile (20:80, v/v) and detected at 270 nm. EE and DLC were calculated according to the below formula:

$$\text{EE }\left(\%\right)=\left(W_{\text{total}}\;-\;W_{\text{free}}\right)/W_{\text{total}}\times 100$$ (1)

$$\text{DLC }\left(\%\right)=\left(W_{\text{total}}\;-\;W_{\text{free}}\right)/W_{\text{liposomes}}\times 100$$ (2)

The accumulative release patterns *in vitro* of free ICT, Lip-ICT and HA-Lip-ICT were assessed via the dialysis technique under controlled conditions at 37°C, involving PBS, pH 7.4, and pH 5.5 PBS buffers containing 0.2% Tween 80 (w/v). In brief, 1.0 mL solutions of free ICT, Lip-ICT and HA-Lip-ICT at the concentration of 5 mg/mL were encapsulated within dialysis bags (MWCO = 3 kDa) and put in the release medium. Subsequently, these dialysis bags were immersed in 50 mL of PBS buffer under gentle agitation at 100 rpm. At predefined intervals (0.5, 1, 2, 4, 6, 8, 10, 12, 24, 36, 48, 60, and 72 h), 1.0 mL release medium was taken out and replaced with same amount of fresh medium. The concentrations of the released ICT were quantified using HPLC. Each sample was replicated in triplicate.

### 2.4 Cellular uptake and binding abilities in HCC cells

To assess the cellular uptake and binding efficacy of HA-Lip in HCC cells, a fluorescent dye called Coumarin 6 (Cou 6) was incorporated within the liposomes. For confocal microscopy analysis, Huh7 and HepG2 cells were seeded onto cell slides at the density of 1 × 10^5^ cells/well. After incubated for 24 h at 37°C, the cells were washed with PBS and 2.0 mL of serum-free DMEM with free Cou 6, Lip-Cou 6 and HA-Lip-Cou 6 (final Cou 6 concentration at 100 ng/mL) were added to each well, free HA was used to block the interaction between HA-Lip-ICT and CD44 Following 1 h of incubation at 37°C, the cells were washed with PBS, fixed using 4% paraformaldehyde, and treated with DAPI for cell nucleus staining. Fluorescence images were taken using an LSM710 laser confocal microscope (Zeiss, Germany).

For the assessment of cellular uptake via flow cytometry, Huh7 and HepG2 cells were seeded at a density of 2 × 10^5^ cells/well in 6-well culture plates and incubated overnight at 37°C. The cells were then given serum-free medium containing 100 ng/mL of free Cou6, Lip-Cou6, and HA-Lip-Cou6 at a final concentration, followed by 1 h of incubation at 37°C. Afterwards, the cells were washed 3 times with PBS, detached using trypsinization, and collected in 0.5 mL of PBS. The mean fluorescence intensity of Cou 6 was measured using a FACScan flow cytometer (BD FACSCalibur, United States).

### 2.5 *In vitro* cytotoxicity analysis

The potential cytotoxic effects of free ICT, empty Lip, Lip-ICT and HA-Lip-ICT on Huh7 cells, HepG2 cells and L02 cells were assessed using the MTT colorimetric assay. Initially, Huh7, HepG2 and L02 cells (1.0 × 10^4^ cells/well) were seeded into 96-well plates and cultured at 37°C with 5% CO_2_ overnight. Subsequently, the cells were exposed to various concentrations (ranging from 0 to 50 μM) of free ICT, Lip-ICT and HA-Lip-ICT, followed by an additional 48 h of incubation. Empty Lip or 0.25% DMSO (vehicle control, VC) was used as control. After removing the medium (100 μL), a solution of MTT dye (15 μL) was added and incubated in the dark for 4 h. The supernatant was abandoned and DMSO (100 µL) was added to dissolve the formazan crystals. Optical density measurements were conducted using a microplate reader at 490 nm. Cell viability was calculated and the values for the 50% and 20% inhibitory concentrations (IC_50_ and IC_20_) were determined. The chemotherapeutic drug 5-fluorouracil (5-Fu) was used as a positive control in this experiment.

### 2.6 Apoptosis test

The assessment of apoptosis for free ICT, Lip-ICT and HA-Lip-ICT in Huh7 and HepG2 cells was carried out using the FITC-Annexin V/propidium iodide (PI) method. In brief, Huh7 and HepG2 cells were seeded into 6-well plates at a density of 5.0 × 10^4^ cells/well and then cultured at 37°C with 5% CO_2_. After incubated overnight, the cells were treated with free ICT, Lip-ICT and HA-Lip-ICT at IC_50_ values of free ICT for another 48 h of incubation with saline as control. Subsequently, the cells were harvested and stained with Annexin V-FITC and PI for 15 min. The analysis of cell apoptosis was conducted using flow cytometry.

### 2.7 Cell cycle analysis

Huh7 and HepG2 cells were cultured in 6-well plates at a density of 5.0 × 10^4^ cells/well and maintained at 37°C with 5% CO_2_. Following overnight incubation, non-cytotoxic doses of free ICT, Lip-ICT and HA-Lip-ICT corresponding to the IC_20_ value of free ICT were added to the Huh7 and HepG2 cells with saline as control. The cells were then further incubated for 48 h. Subsequently, the cells were collected, rinsed with PBS, and fixed using 70% ethanol. The fixed cells were stored at 4°C overnight. Then, Rnase A was introduced and incubated for 45 min at 37°C, followed by PI staining in the dark for 30 min. The distribution of cell cycle phases was analyzed using flow cytometry with total of 50000 events.

### 2.8 Acute toxicity analysis of HA-Lip-ICT in mice

To assess potential *in vivo* toxicity and provide guidance for evaluating anti-tumor effectiveness, an acute toxicity study of HA-Lip-ICT was conducted in normal Kunming mice by a single intravenous injection of the tested formulations, with the treated mice being observed for 7 days. After an adaptation period, the animals were randomly divided into six groups (n = 5), which included 5-Fu (125 mg/kg), empty Lip, ICT (5 mg/kg), Lip-ICT (5 mg/kg) and HA-Lip-ICT (5 mg/kg), while saline was used as the negative control and 5-Fu served as the positive control. All mice were injected 0.2 mL of various formulations and their body weights were measured every day. The levels of creatine kinase (CK), lactate dehydrogenase (LDH), alanine aminotransferase (ALT), aspartate aminotransferase (AST), creatinine (CREA) and blood urea nitrogen (BUN) were assessed through automated analysis using a biochemistry analyzer (ADVIA 2400, Siemens, Munich, Germany) in the Affiliated Hospital of Southwest Medical University (Luzhou, China). On the 7th day following treatment, the mice were sacrificed. The heart, liver, spleen, lungs, and kidneys were promptly removed, rinsed with saline and then fixed in 10% formalin and stained for hematoxylin and eosin (H&E) pathological analysis.

### 2.9 Distribution of HA-Lip-ICT in Huh7 tumor-bearing mice

A Huh7 tumor-bearing nude mice model was induced by subcutaneously injecting 0.1 mL of Huh7 cell suspension (2.5 × 10^6^ cells) into the right flank of female BALB/c nude mice aged 6–8 weeks. Once the tumors reached a mean size of 100 mm^3^, mice were separated into two groups (n = 5) and were administered DiR-encapsulated liposomes (Lip-DiR) and HA-modified DiR-loaded liposomes (HA-Lip-DiR) via tail vein injection at a dose of 500 μg/kg DiR. Fluorescence imaging was conducted at 3, 8, and 12 h post-injection using an IVIS imaging system (Kodak, PerkinElmer, Waltham, MA, United States). Subsequently, the mice were sacrificed, and the major organs and tumors were removed for *ex vivo* fluorescence imaging.

### 2.10 *In vivo* antitumor efficacy

Huh7 tumor-bearing nude mice were established following the aforementioned procedure (Zhuo et al., 2015). Once the tumor size reached 100 mm^3^, the mice were divided randomly into 5 groups (5 mice per group), each containing five mice. Subsequently, intravenous administrations of saline and different ICT (5 mg/kg) formulations were administered every 2 days for a total of 7 doses. Tumor volume measurements were taken at 2-day intervals by measuring both the longest axis (L) and the shortest axis (W) of the tumor using a vernier micrometer. On day 14, all the animals were euthanized, and the tumors were retrieved, and their weights were recorded. The anti-tumor activity was assessed through tumor growth inhibition (TGI) by measuring the mean tumor weight (MTW) of the treated groups (TG) compared to the saline group (CG). Both tumor volume and TGI were calculated with the below formula:

$$\text{Tumor volume }\left({\text{mm}}^{3}\right)=½\;\frac{1}{2}\left(L\times W^{2}\right).$$ (3)

$$\text{TGI }\left(\%\right)=\left({\text{MTW}}_{\text{TG}}\;-\;{\text{MTW}}_{\text{CG}}\right)\;/\;{\text{MTW}}_{\text{CG}}\times 100.$$ (4)

The tumor tissues were then subjected to a TUNEL assay to evaluate tumor cell apoptosis, Ki-67 staining for tumor cell proliferation assessment, and immunohistochemistry AFP staining to measure HCC treatment efficacy (H. Li et al., 2021).

### 2.11 Statistical analysis

All results were performed in triplicate independent experiments and data were represented as mean ± SD. A student’s t-test or one-way analysis of variance (ANOVA) were applied to test for significance in the experiments. The statistical differences were considered significant at *p* < 0.05 and very significant at *p* < 0.001.

## 3 Results

### 3.1 Preparation and characterization of HA-Lip-ICT

**FIGURE 1:**
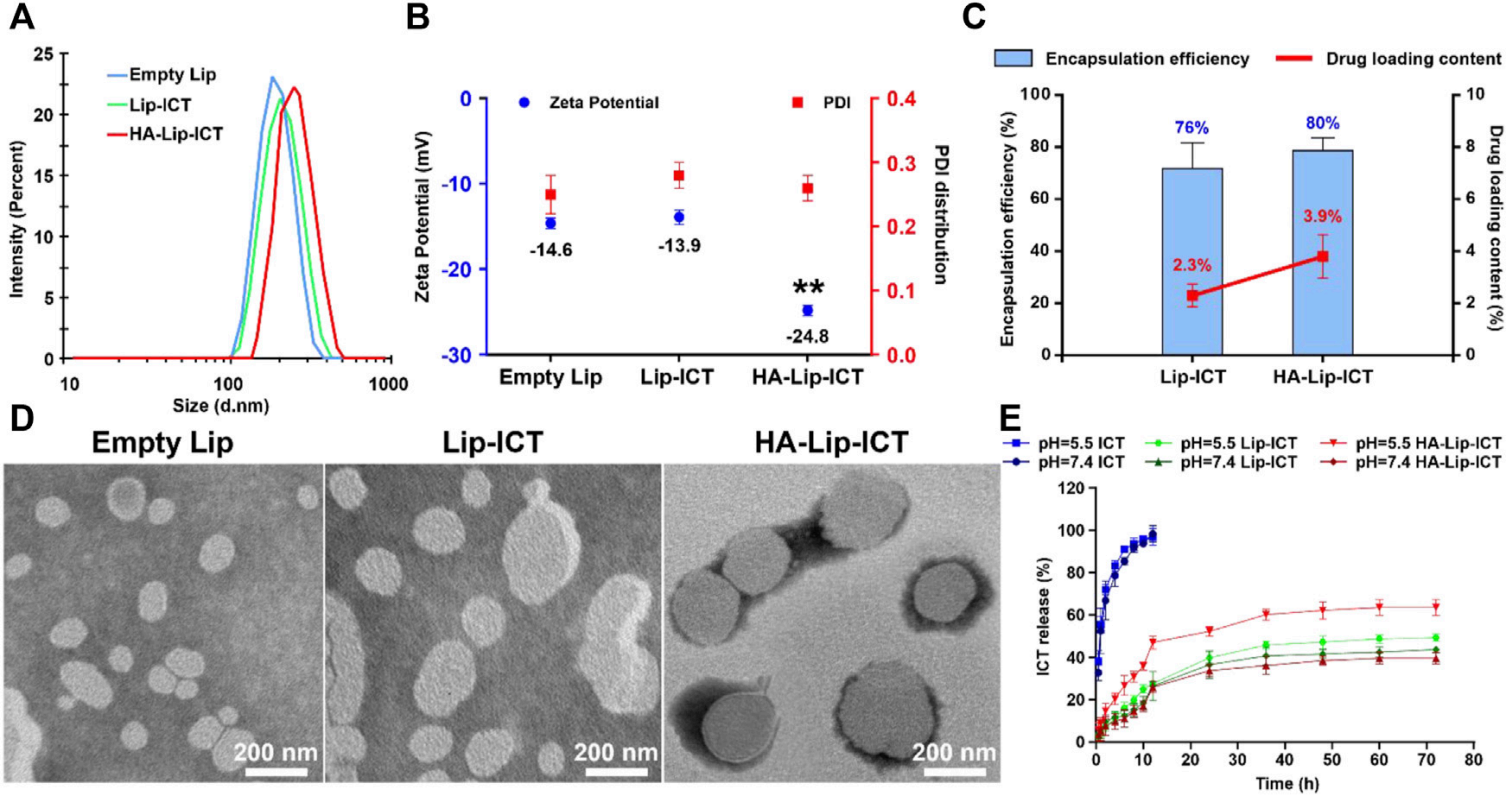
Characterization of empty Lip, Lip-ICT and HA-Lip-ICT, including **(A)** distribution of the hydrodynamic size detected by DLS, **(B)** zeta potential and PDI distribution, **(C)** encapsulation efficiency and drug loading content, **(D)** TEM micrographs, and **(E)** accumulative release profile of free ICT *in vitro* and ICT from Lip-ICT and HA-Lip-ICT in pH 7.4 and pH 5.5 PBS at 37°C during 72 h. Data are shown as mean ± SD (n = 3). **p < 0.01.

The synthesis of hyaluronic acid-modified cholesterol (HA-Chol) was confirmed using NMR (Supplementary Figures S2–4) The particle size, PDI, zeta potential, encapsulation efficiency and drug loading content were evaluated. The obtained results indicated that the particle size of all formulations demonstrated within the range of approximately 160–200 nm, with PDI values less than 0.3 (Figures 1A,B). The zeta potential of HA-Lip-ICT was observed to decrease to −24.8 ± 0.36 mV (Figure 1B), which is the most negatively charged. It can be seen from the FTIR spectra (Supplementary Figure S5) that HA-Lip, ICT and HA-Lip-ICT exhibited wide bands around 3268.81, 3407.32, and 3420.661 cm^−1^, respectively, which have been confirmed from the O-H bonds stretching (Pawlikowska-Pawlęga et al., 2013). In ICT, the C=O and C–O bonds vibrations led to the absorbance of 1638.73 and 1399.34 cm^−1^, respectively. When ICT was incorporated within liposomes, those peaks shifted toward smaller wave numbers (1628.36 and 1396.15 cm^−1^, respectively). The encapsulation efficiencies for HA-Lip-ICT and Lip-ICT were 80% and 76%, respectively. The drug loading contents were 3.9% and 2.3%, respectively (Figure 1C). Morphological examination through TEM revealed that both HA-Lip-ICT and Lip-ICT exhibited spherical shapes with a uniform size distribution, while no aggregation occurred (Figure 1D). Cumulative release of ICT from Lip-ICT and HA-Lip-ICT were investigated using a dialysis method at 37°C and the resulting release curves were depicted in Figure 1E. Comparing with free ICT, both Lip-ICT and HA-Lip-ICT displayed a 2-phase release pattern with a relatively fast initial release then a slow sustained release phase. Within the first 12 h, approximately 36.6% of ICT was released from Lip-ICT, while this proportion was approximately 29.8% for HA-Lip-ICT under pH 7.4 conditions. After 24 h, the accumulated release quantities of ICT from HA-Lip-ICT and Lip-ICT were 45.8% and 63.6% under pH 5.5 conditions, respectively.

The storage stability of HA-Lip-ICT was assessed at 4°C (Supplementary Table S1) and 25°C (Supplementary Table S2), respectively. The particle size and PDI of HA-Lip-ICT did not change significantly after 10 days at 4°C, and almost no drug was released at that temperature. In contrast, the particle size and PDI increased significantly and the EE declined after storage at 25°C for 5 days. These results suggest that HA-Lip-ICT are stable only at low temperatures and for a limited time.

### 3.2 Cellular uptake and binding abilities in HCC cells

**FIGURE 2:**
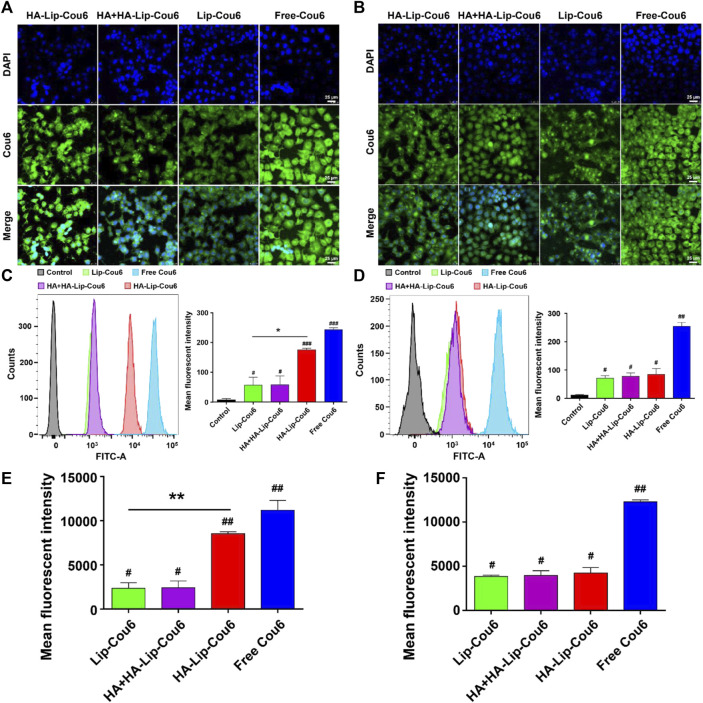
Cellular uptake abilities of free Cou 6, Lip-Cou 6, HA + HA-Lip-Cou 6 and HA-Lip-Cou 6 on Huh7 cells and HepG2 cells post incubation at 37°C for 1 h. Concentration of Cou 6 was 100 ng/mL. Laser scanning confocal microscopy images of **(A)** Huh7 cells and **(B)** HepG2 cells treated with different formulations. Blue and green indicate the fluorescence of DAPI and Cou 6. Cou 6 uptake in **(C)** Huh7 cells and **(D)** HepG2 cells was determined and the semi-quantitative analysis of Cou 6 uptake based on flow cytometry was plotted. **(E, F)** Quantitative analysis of Cou 6 uptake based on confocal microscopy. Each bar shows the average fluorescence intensity with standard deviation (n = 3), *p < 0.05, **p < 0.01, #*p* < 0.05, ##*p* < 0.01, ###*p* < 0.001 vs. control group.

As depicted in Figure 2, uptake rate in HA-Lip-Cou 6 group exhibited an approximately two-fold increase compared with the Lip-Cou 6 group in Huh7 cells while there were no significant changes in HepG2 cells. The uptake rate in HA + HA-Lip-Cou 6 group was decreased to approximately 63% to HA-Lip-Cou 6 group in Huh7 cells while there were no notable changes in HepG2 cells. The confocal microscopy pictures in Figures 2A,B and their quantification results in Figures 2E,F yielded similar outcomes correlated to the data from flow cytometry in Figures 2C,D. Specifically, the considerably intensified green fluorescence in the HA-Lip-Cou 6 group compared with Lip-Cou 6 group confirmed a significant enhancement in cellular uptake. After CD44 receptor blocking through pre-treatment using free HA, the intracellular Cou 6 intensity in the HA-Lip-Cou 6 group exhibited a remarkable reduction.

### 3.3 *In vitro* cytotoxicity assay

**FIGURE 3:**
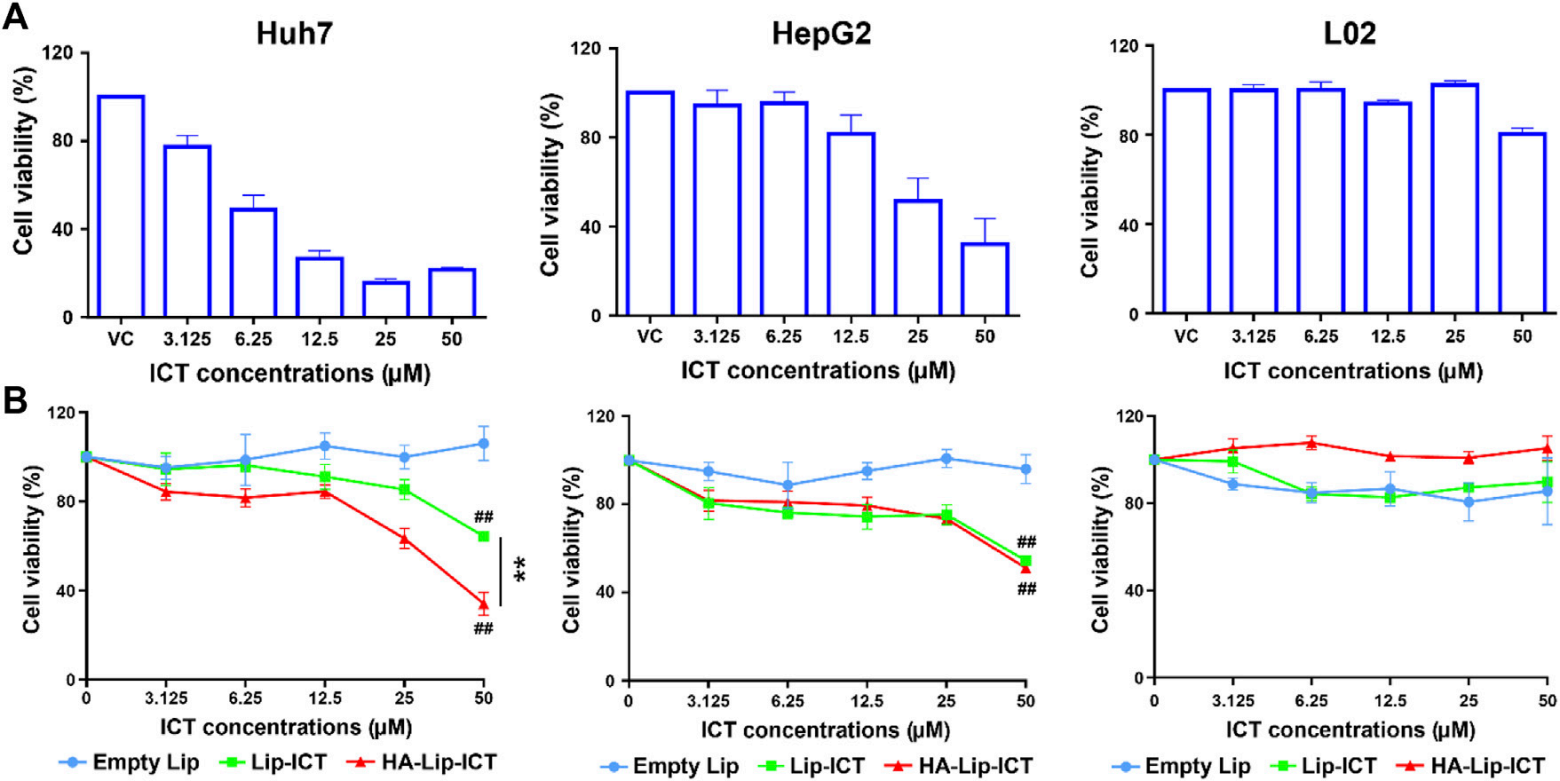
Cell viability of Huh7, HepG2 and L02 cells after incubation with **(A)** free ICT and **(B)** different liposome formulations at various concentrations for 48 h **p < 0.01, ##*p* < 0.01 vs. control group.

Cell viability was evaluated using an MTT assay to assess the cytotoxicity of two different ICT liposomes and free ICT. The survival rates of Huh7 cells, HepG2 cells and L02 cells are presented in Figure 3. The growth inhibitory effects of the ICT formulations exhibited a dose-dependent pattern. Among these, free ICT displayed the highest cytotoxicity at 48 h, with IC_50_ values of 8.4 ± 0.8 μM and 28.6 ± 8.4 μM in Huh7 cells and HepG2 cells, respectively. Both Lip-ICT and HA-Lip-ICT exhibited growth inhibition on HCC cells. Specifically, in Huh7 cells, the IC_50_ values were 34.2 μM and >50 μM for HA-Lip-ICT and Lip-ICT, respectively. In HepG2 cells, IC_50_ values were both >50 μM, respectively. Significantly, HA-Lip-ICT demonstrated greater cytotoxicity than Lip-ICT. The drug 5-Fu at 0.19 μM, which was used as a positive control, demonstrated around 70%–80% of cytotoxicity on HCC cell lines. In addition, HA-Chol showed no cytotoxicity on Huh7, HepG2 or L02 cells (Supplementary Figure S6), which correlated with the previous study (Song et al., 2019).

### 3.4 Cell apoptosis induction and cell cycle arrest study

**FIGURE 4:**
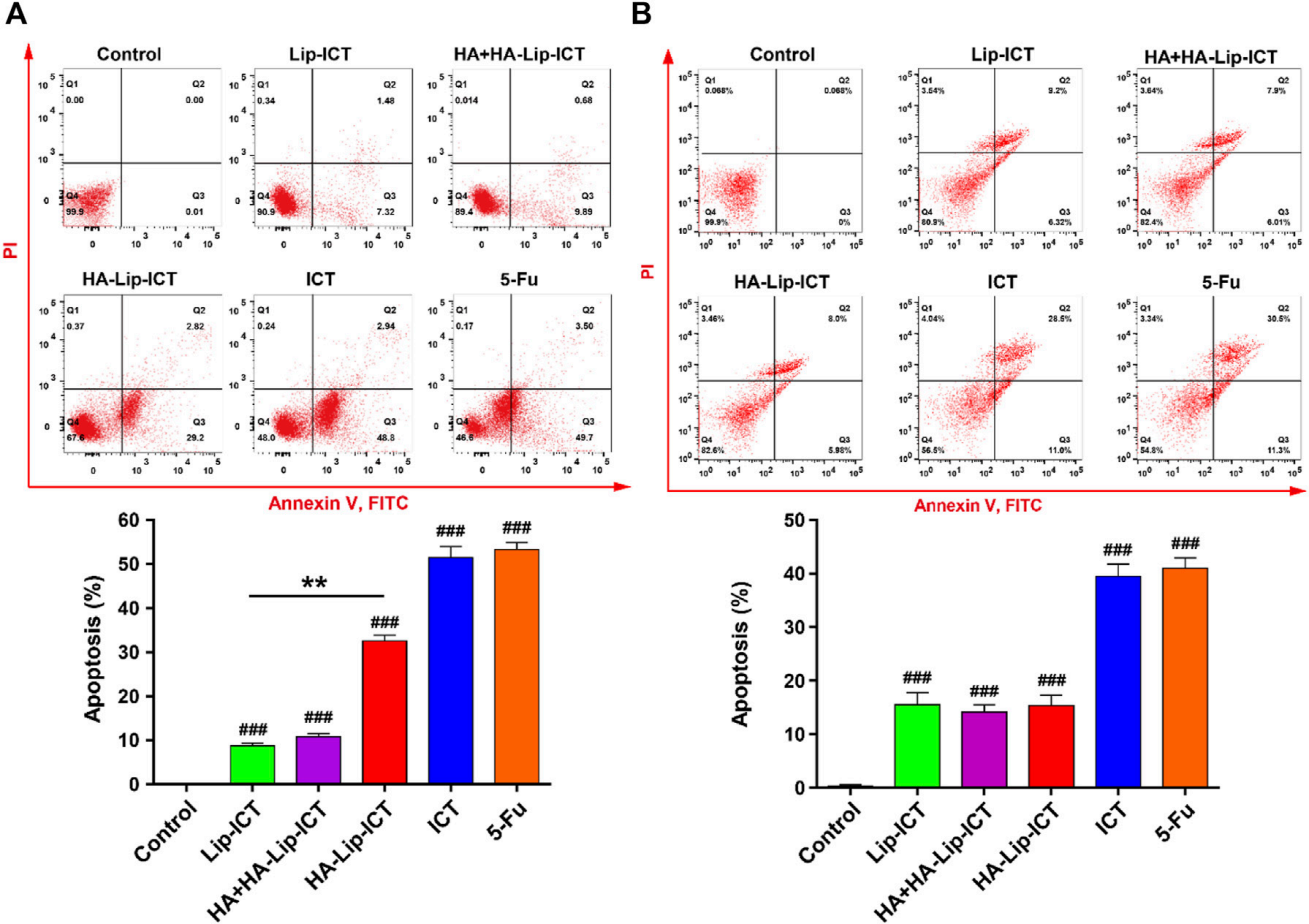
Effect of ICT in various Lip-ICT formulations on apoptosis induction of **(A)** Huh7 cells and **(B)** HepG2 cells after 48 h of treatment. The chemotherapeutic drug 5-Fu was used as positive control. Data are represented as mean ± SD, n = 3. **p < 0.01, ###*p* < 0.001 vs. control group.

Apoptosis induction in Huh7 and HepG2 cells by free ICT, Lip-ICT and HA-Lip-ICT was assessed using Annexin V-FITC and PI staining. The chemotherapeutic drug 5-Fu used was as a positive control. The HA-Lip-ICT group demonstrated a three-fold increase in the apoptosis ratio compared with the Lip-ICT group in Huh7 cells (Figure 4A), while there was no significant change between these two groups observed in HepG2 cells (Figure 4B).

**FIGURE 5:**
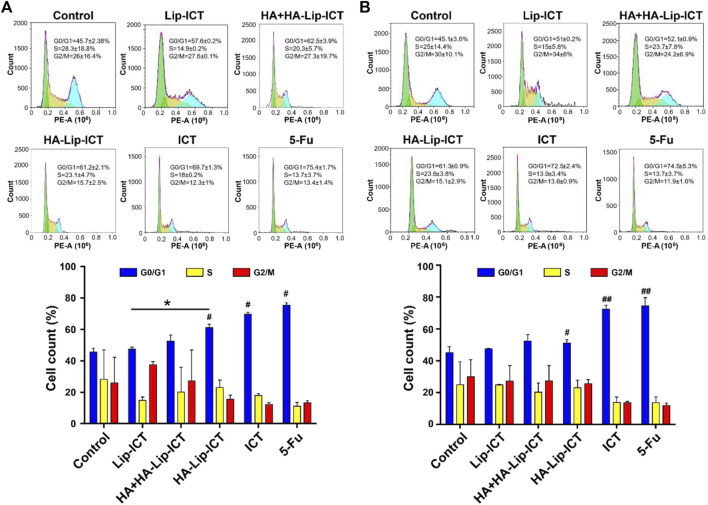
Effect of ICT in various Lip-ICT formulations on cell cycle progression of **(A)** Huh7 cells and **(B)** HepG2 cells after 48 h of treatment. The chemotherapeutic drug 5-Fu was positive control. Data are represented as mean ± SD, n = 3. *p < 0.05; #*p* < 0.05, ##*p* < 0.01 vs. control group.

The apoptosis mechanism of HA-Lip-ICT involved in studying the cell cycle arrest of treated Huh7 and HepG2 cells was further investigated using various formulations. As depicted in Figure 5A, the percentage of cells in the G_0_/G_1_ phase significantly increased to 61% in the HA-Lip-ICT group, compared with 51% in the Lip-ICT group (*p* < 0.05) in Huh7 cells, which correlated with the previous study (S. Wang et al., 2019). However, there was no remarkable change observed in HepG2 cells between these two groups (Figure 5B).

### 3.5 HA-Lip-ICT showed no acute toxicity *in vivo*

**FIGURE 6:**
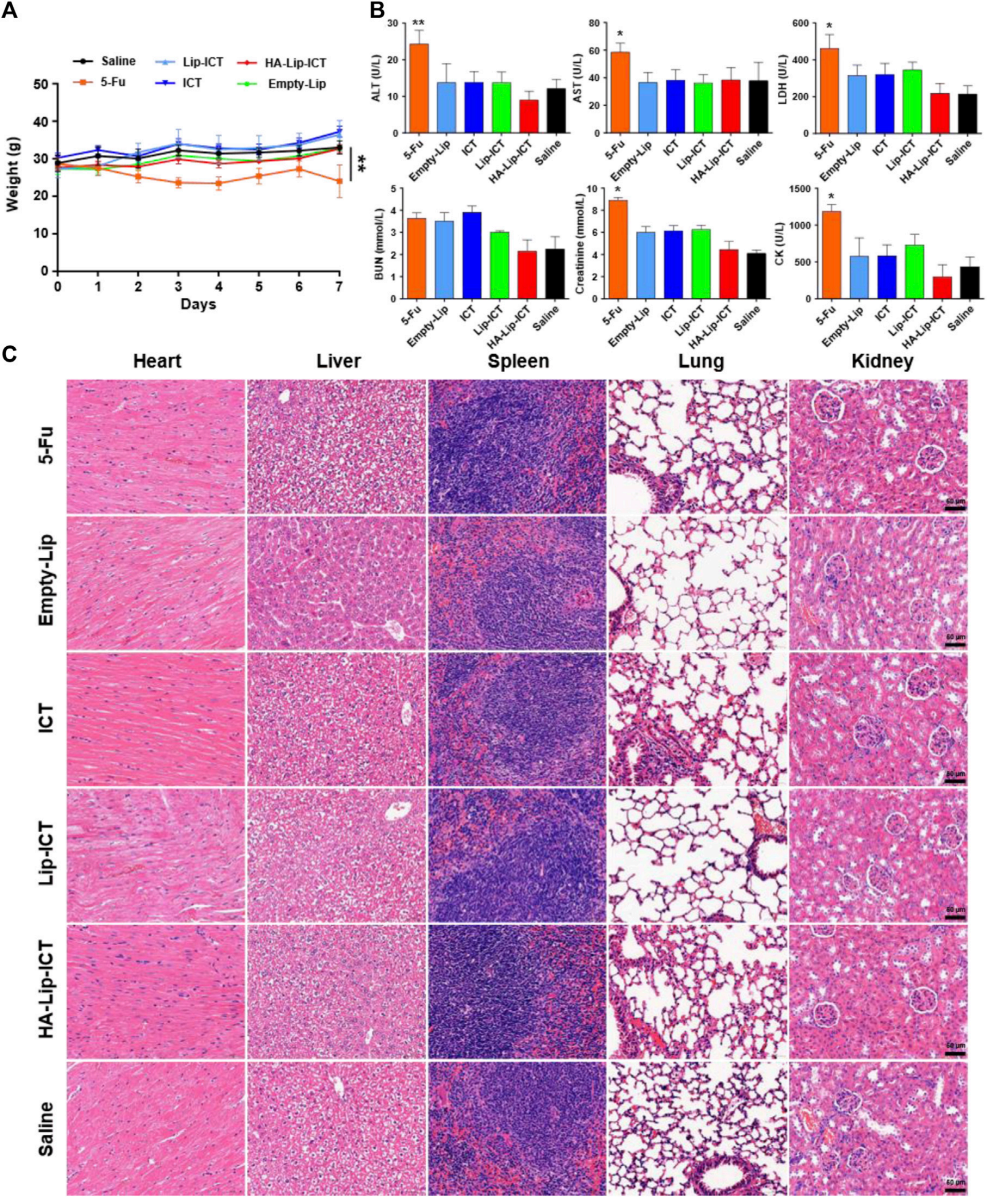
Biosafety of the formulations. **(A)** Body weights of mice after various formulations treatments (n = 5). **(B)** Biochemical analysis of ALT and AST, LDH, BUN, Creatinine and CK in normal mice treated with different formulations (n = 5). ALT: alanine aminotransferase; AST: aspartate aminotransferase; LDH: lactate dehydrogenase; BUN: blood urea nitrogen; CK: creatine kinase. **(C)** H&E-staining images of major organs post treatment. *p < 0.05, **p < 0.01.

The *in vivo* toxicity of HA-Lip-ICT was assessed in normal Kunming mice. Following treatment with various formulations, all treated mice exhibited a steady increase in body weight, except for the 5-Fu group (Figure 6A), wherein mice treated with 5-Fu showed a significant weight decrease of 11.9% (*p* < 0.01). Furthermore, serum biochemical analyses were conducted to evaluate the marker of liver function (ALT and AST), heart function (CK and LDH) and kidney function (Creatinine and BUN) (Figure 6B). Apart from 5-Fu treated group, serum biomarker analysis results for other groups fell inside the healthy range, showing no remarkable differences. After treated with 5-Fu, ALT and AST levels notably increased when compared with saline treated group (*p* < 0.05). As for kidney function marker (BUN), all treatment groups exhibited normal data without obvious differences when compared with saline treated group (*p* > 0.05). Finally, histological examination of tissue sections through H&E staining revealed no typical necrosis, including nuclear fragmentation, shrinkage, or dissolution in normal mice, indicating there were no obvious pathological alterations in the major organs across all groups (Figure 6C).

### 3.6 Biodistribution *in vivo*

**FIGURE 7:**
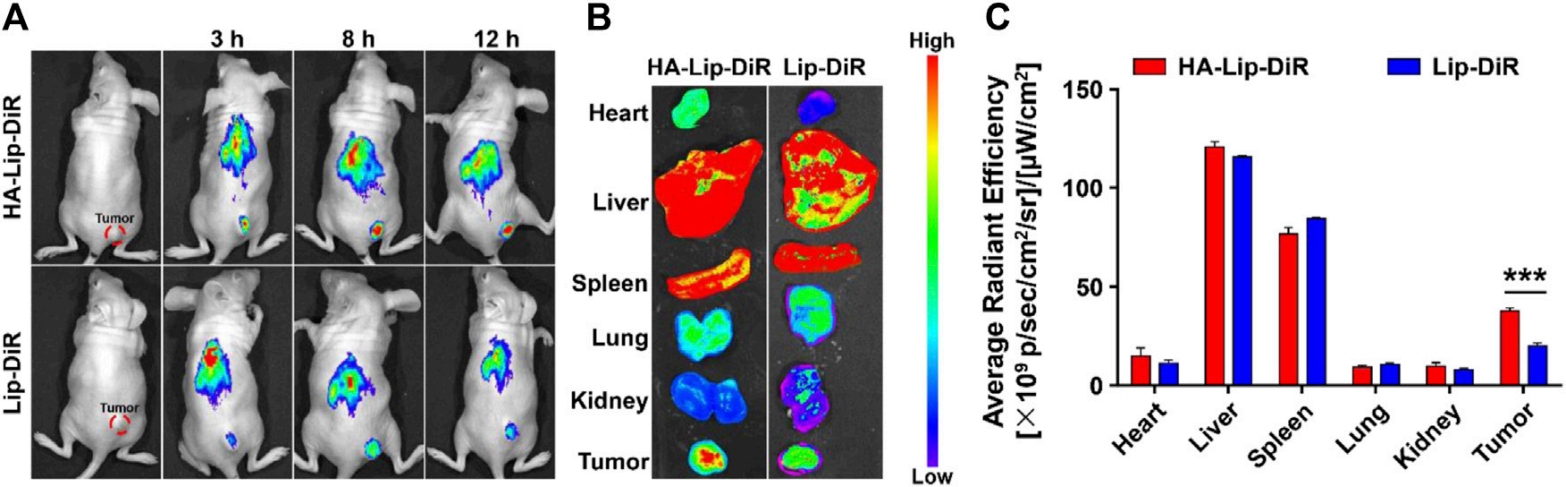
*In vivo* targeting of DiR-loaded Lip and HA-Lip liposomes to nude mice bearing Huh7 tumors by NIRF imaging. **(A)** *In vivo* fluorescence images of mice at different time points after treatment, **(B)** Images of dissected tumors and organs taken *ex vivo* 12 h after injection. **(C)** Semi-quantitative analysis of the *ex vitro* fluorescence intensity. Data are shown as mean ± SD (n = 6). ***p < 0.001.

The distribution *in vivo* and tumor-binding efficacy of formulations were measured using near-infrared fluorescence imaging (Figure 7A). As early as 3 h after the administration of HA-Lip-DiR, a clear fluorescence signal was found in the tumor tissue. This signal continued to increase, reaching its peak at 8 h, and remained visible throughout the 12-h monitoring period. Conversely, the Lip-DiR treated group exhibited just a faint fluorescence signal at the location of tumor after 3 h. Throughout the imaging duration, HA-Lip-DiR demonstrated substantially superior and more sustained accumulation in the tumor tissue compared with Lip-DiR. The *ex vivo* fluorescence images of removed tumors (Figure 7B) corroborated the findings from the *in vivo* imaging. Quantitative analysis of fluorescence intensity revealed that the tumor fluorescence intensity of HA-Lip-DiR was 1.9-fold greater than that of Lip-DiR (Figure 7C).

### 3.7 Efficacy against tumor *in vivo*

**FIGURE 8:**
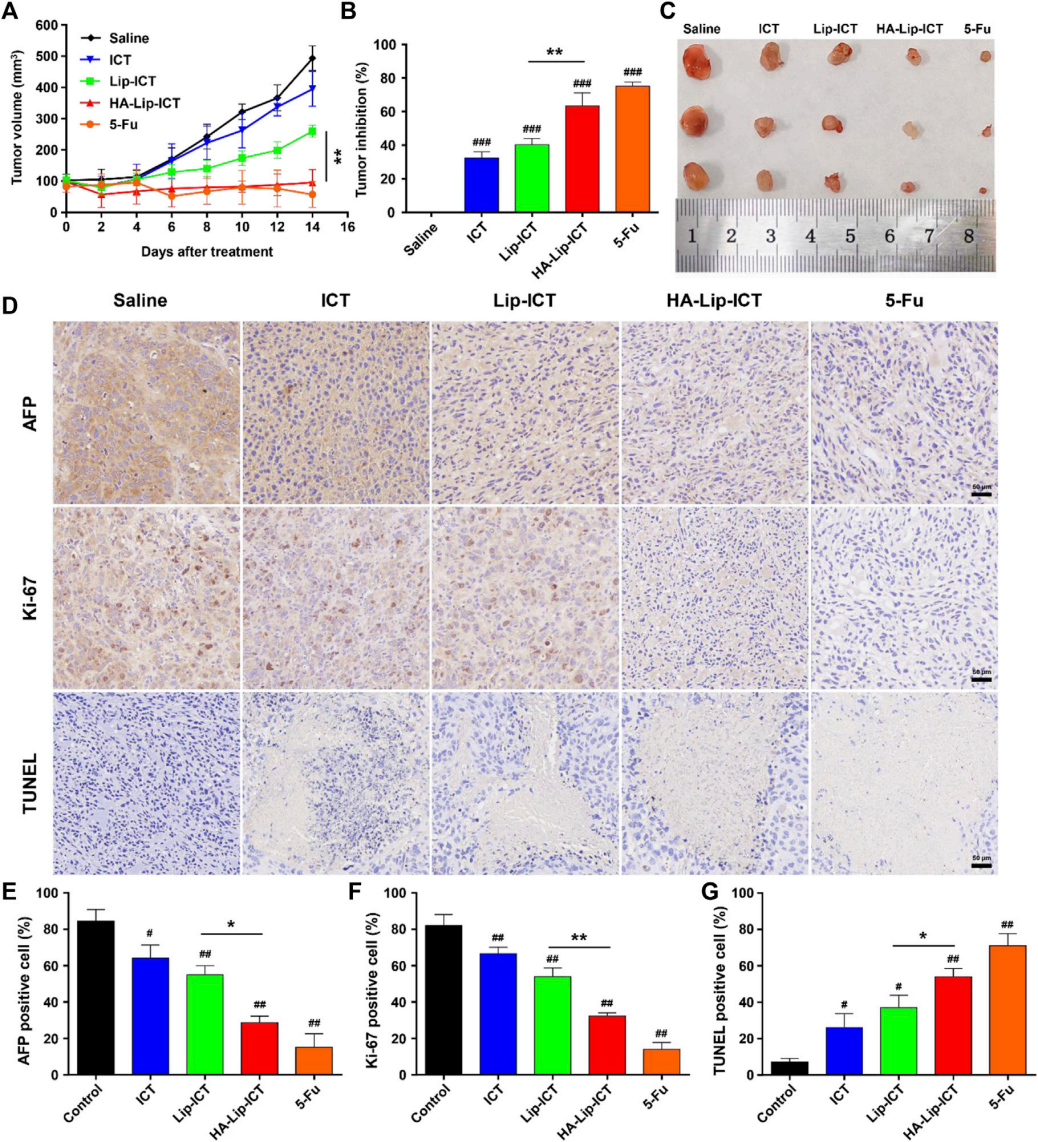
Anti-tumor effect of HA-Lip-ICT on Huh7 xenograft model. **(A)** The tumor volume and **(B)** tumor-growth inhibition rate were measured and **(C)** the representative images of tumor tissue from each group were presented. *p < 0.05, **p < 0.01, #*p* < 0.05, ##*p* < 0.01, ###*p* < 0.001 vs. control group (n = 5). **(D)** AFP, Ki-67 and TUNEL images of tumor tissues and their quantification results **(E–G)**. The brown colors in the images indicate AFP-positive, TUNEL-positive or Ki-67-positive cells.

The anti-tumor effectiveness is showed in Figure 8, wherein the different ICT formulations demonstrated a notable degree of tumor inhibition compared with the fast tumor growth observed in the saline-treated group. In comparison to the saline group, the free ICT, Lip-ICT, and HA-Lip-ICT groups exhibited tumor growth inhibition rates of 32.5%, 40.4%, and 63.4%, respectively. Notably, HA-Lip-ICT exhibited superior antitumor efficacy compared with all other ICT treatments. The HA-Lip-ICT group exhibited significantly smaller average tumor sizes and increased tumor inhibition rates (Figures 8A–C), further confirming its enhanced antitumor efficacy.

To assess the antitumor efficacy at the cellular level, the results were showed in (Figure 8D) and their quantification results (Figures 8E–G). Local tumor cell apoptosis was evaluated using the TUNEL staining assay. The HA-Lip-ICT group showed a higher dUTP-positive rate when compared with other groups, consistent with the tumor volume inhibition results. Ki-67, a marker of cell proliferation, was significantly downregulated in mice treated with 5-Fu and HA-Lip-ICT, indicating that HA-Lip-ICT successfully suppressed the growth of cancer cells *in vivo*, similar to 5-Fu. Moreover, the AFP-positive area, which reflects oncofetal protein expression, was notably reduced in animals treated with 5-Fu and HA-Lip-ICT. This result suggests a greater curative effect on HCC, which was observed in Huh7 xenografts following treatment with 5-Fu and HA-Lip-ICT.

## 4 Discussion

Icaritin exhibits a multifaceted mechanism of action against liver cancer, including inducing apoptosis through a caspase-dependent pathway, inhibiting cell proliferation by blocking the cell cycle and inducing cell cycle arrest, modulating various signaling pathways such as PI3K/AKT, MAPKs, and JAK/STATs, inhibiting tumor angiogenesis by suppressing VEGF and other angiogenic factors, enhancing the immune response by increasing cytokine production and activating immune cells, inhibiting the growth and self-renewal of cancer stem cells, and modulating the tumor microenvironment by suppressing pro-inflammatory cytokines and promoting immune cell infiltration (Yang et al., 2019; Yin et al., 2020; Zhang et al., 2020; Lu et al., 2022; Reyes-Hernández et al., 2024). Despite its potential, the clinical application of icaritin for cancer treatment is currently limited by its low water solubility and restricted bioavailability.

Hyaluronic acid-modified liposomes have shown promise in targeted drug delivery for cancer. These liposomes can be designed to target CD44-overexpressing cancer cells, which are prevalent in lung and colorectal cancers. Studies have shown their effectiveness in delivering ursolic acid to lung cancer cells, co-delivering doxorubicin and paclitaxel, and carrying 5-fluorouracil to colorectal cancer cells. This versatility highlights the potential of this technology for various cancer drug delivery applications (Song et al., 2019; Mansoori et al., 2020; Ma et al., 2023).

To boost the anti-tumor efficacy of icaritin (ICT) on liver cancer, hyaluronic acid (HA)-modified liposomes (Lip) were utilized. The tumor-specific effect of ICT can be achieved by active targeting via the receptor-ligand interaction between HA and CD44 plus passive targeting by accumulation around tumors due to the Enhanced Permeability and Retention (EPR) effect. The prepared HA-Lip-ICT displayed a spherical shape with a narrow size distribution fell in the range of 160–200 nm. The slightly increased particle size compared with non-modified liposomes suggested successful HA coverage on the liposome surface. Earlier investigations have indicated that nanoparticles with smaller sizes (<200 nm), less than the pore size of leaky vasculatures and hindered lymphatic drainage, are conducive to the accumulation and localization of nanoparticles at solid tumor sites through the enhanced permeability and retention (EPR) effect (Yang et al., 2007; Duan et al., 2013; Ravar et al., 2016; Lei et al., 2019). Smaller nanoparticles (around 100 nm), especially those with specific structures like PEGylated liposomes, exhibited enhanced resistance to reticuloendothelial system (RES) phagocytosis due to the dense PEG layer on their surfaces (Barua and Mitragotri, 2014). As the presence of carboxyl groups from HA molecules on the surface of HA-modified liposomes, the zeta potential of HA-Lip-ICT was observed to decrease to −24.8 ± 0.36 mV, the reversal in potential of HA-Lip-ICT induces repulsive forces among liposomes, promoting a stable system and preventing aggregation or deposition on vessel walls. In contrast to non-modified liposomes, HA-modified liposomes demonstrated potential in evading RES phagocytosis, thus extending circulation time in the bloodstream (Song et al., 2019). So, hydrophilic HA holds promise as an alternative to PEG molecules in achieving prolonged circulation in liposomes. The FTIR result indicated that C=O and C–O groups of ICT participated in hydrogen bonds formation with soybean lecithin of liposomes due to their peaks shifted toward smaller wave numbers, which confirmed the successfully incorporated of ICT (Zhou et al., 2014).

The release outcomes in normal physiological pH of the bloodstream at pH 7.4 can be attributed to the presence of an HA layer on the liposome outer layer, which contributed to the delayed release of ICT from the liposomes. Upon wrapping the liposomes with HA, the HA molecules rapidly adsorb water in an aqueous environment, causing them to expand and create a dense hydration membrane on the shell of liposomes. This process reduces the phospholipid bilayer’s fluidity and permeability (Ossipov, 2010; Tiantian et al., 2014). The compact, water-attracting HA structure encasing liposomes act like a barrier, limiting dispersion of ICT from the liposomes into the media. The gradual release profile exhibited by HA-Lip-ICT demonstrates its exceptional stability, preventing fast ICT release in the bloodstream and promoting the accumulation of liposomes at tumor sites. While the acidic environment of the tumor at pH 5.5, the observation could be attributed to the instability of the liposome structure. HA is susceptible to hydrolysis in an acidic environment, causing the polymer chains to undergo random scission, ultimately degrading the integrity of the liposome structure (Kim et al., 2012; Nascimento et al., 2016). Another possible explanation is that the hydrolysis of HA exposes the lipid bilayer, while the cholesterol segment within the HA-Chol conjugate remains embedded in the bilayers. Excessive cholesterol content can undermine bilayer stability. Consequently, the loss of the protective hydrophilic layer enhances the phospholipid bilayer’s fluidity and permeability, facilitating the release of the drug from the liposomes (Song et al., 2019).

As for the cellular uptake results, given that HA-Lip-Cou 6 and Lip-Cou 6 shared similar constituents, except for the presence of HA, the distinction in uptake could be directly attributed to HA. The primary uptake route of HA-Lip-Cou 6 involves CD44 facilitated endocytosis. Previous study also postulated that augmentation in cellular uptake could be attributed to the presence of CD44 receptors on the cell surface (Misra et al., 2015). So, the superior uptake level of HA-Lip-Cou 6 may come from the higher expression level of CD44 in Huh7 cells than in HepG2 cells (Supplementary Figure S7) (Cannito et al., 2022). Previous research has suggested that the existence of rivaling HA within the growth medium reduces the cellular uptake of HA-conjugated nanoparticles. In order to support this idea, a receptor competition experiment was created (Tran et al., 2014). The heightened cell uptake rate induced by HA coating was notably decreased when there is free HA, suggesting a competitive interaction between free HA and HA-Lip-Cou 6 for the same CD44 receptor. This outcome indicated the pivotal role of the HA-CD44 interaction in facilitating enhanced cellular uptake. These findings suggested heightened uptake by cells observed with HA-Lip-Cou 6 is facilitated by the HA-CD44 binding between the liposomes and Huh7 cells. However, the main findings are based solely on one CD44 high HCC cell line (Huh7), and a second CD44-high HCC cell line or a CD44 knockout line of Huh7 cells and a CD44-overexpressing line of HepG2 maybe needed to be used to provide more robust evidence for the efficacy of HA-Lip-ICT.

*In vitro* cytotoxicity results, the IC_50_ level of ICT in Huh7 cells is much lower than that in HepG2 cells, this different effect might be attributed to its rapid cellular uptake through passive diffusion and an optimum concentration in Huh7 cells according to previous studies (Wang et al., 2020a; Liu et al., 2021). While the enhanced cytotoxicity of HA-Lip-ICT could be attributed to the increasing cellular uptake of ICT facilitated by HA-CD44-mediated endocytosis in Huh7 cells.

The apoptosis results suggested that HA-Lip-ICT enhanced the apoptosis-inducing capacity of ICT, particularly by targeting CD44 overexpressed cells. Additionally, the apoptosis ratio of the HA-Lip-ICT group was lower than that of the free ICT group. This could be attributed to the rapid cellular uptake of free ICT via passive diffusion, which might not involve sustained release mechanisms (Wang et al., 2020a; Liu et al., 2021). Furthermore, the elevated G_0_/G_1_ phase in the HA-Lip-ICT group could potentially be attributed to the promotion of internalization through HA-CD44 interactions. The potential mechanisms of HA-Lip-ICT may be the generation of reactive oxygen species (ROS), which damage cellular components and disrupt cellular processes, triggering apoptosis and cell cycle arrest (Ma et al., 2023). Additionally, upregulation of p53 and apoptosis-related proteins in the transforming growth factor-β signaling (ARTS) pathway, leading to cytochrome-c release, caspase-3 activation, and mitochondrial apoptosis (Ma et al., 2023). Furthermore, causing cell cycle arrest at the G_0_/G_1_ phase, inhibiting cell proliferation and promoting apoptosis (Mansoori et al., 2020).

The *in vivo* biosafety findings suggest that both ICT and liposomes did not cause significant systemic toxicity in healthy mice. These findings suggested that the treatments did not lead to noticeable hepatic, cardiac or renal disorders. And the H&E result further confirms the favorable biocompatibility and biosafety of ICT or various liposomes in this study.

These *in vivo* biodistribution outcomes highlight the significant enhancement in liposome delivery to tumors achieved through HA modification. Hydrophilic HA coating on HA-Lip-DiR contributed to enhanced serum stability, preventing fast non-specific clearance by the reticuloendothelial system (RES) organs, such as spleen and liver. So, it could extend the circulation time in blood (Liang et al., 2015; M; Zhang et al., 2021). Moreover, HA functionalization facilitated targeted binding and internalization at the tumor site through receptor-ligand interacted endocytosis for HA-Lip-DiR. This targeted binding allowed for extensive penetration into the tumor tissue, surpassing the effects of enhanced permeability and retention (EPR) alone.

The best *in vivo* anti-tumor effect of HA-Lip-ICT can be attributed to both the passive targeting mediated by the EPR effect and the active targeting facilitated by the interaction between HA and CD44, as well as the accelerated release of ICT caused by the faint acidic tumor microenvironment (Song et al., 2019; Wang et al., 2020b). Furthermore, this study demonstrated that even at a relatively low dose (5 mg/kg), the therapeutic effectiveness of ICT *in vivo* for liver cancer was prominent after encapsulation into HA-coated liposomes. The exceptional anti-tumor effect can be credited to the strong tumor-targeting effectiveness of HA-Lip-ICT, achieved through a combination of passive and active targeted delivery methods. The presence of HA on the surface of liposome improved its selective uptake via HA/CD44 receptor-mediated endocytosis. Therefore, in conjunction with previous research, the potential of ICT highlighted in this study, particularly when delivered through its HA-modified liposomal nanocarrier, indicates a promising approach for liver cancer treatment due to its potent antitumor effects.

## 5 Conclusion

A novel approach was introduced using a nanoplatform of liposomes modified with hyaluronic acid (HA) to enhance the uptake of the antitumor agent ICT for liver cancer. These HA-modified liposomes, featuring a negatively charged surface, displayed high efficiency in encapsulating ICT and had a compact particle size. *In vitro*, it was observed that HA-Lip-ICT significantly improved intracellular uptake, increased cytotoxicity, induced apoptosis, and caused cell cycle arrest when compared to Lip-ICT. This improvement can be attributed to the involvement of HA in facilitating CD44 receptor-mediated endocytosis. Functionalizing with HA greatly enhanced the liposomes’ ability to target tumors. *In vivo*, HA-Lip-ICT demonstrated superior antitumor effectiveness with minimal systemic side effects than free ICT and Lip-ICT due to the faint acidic tumor microenvironment. In summary, HA-Lip-ICT holds promising potential as an effective strategy for targeted drug delivery, enhancing the treatment of liver cancer. Additionally, the anti-tumor mechanism of HA-Lip-ICT requires further investigation.

## Data Availability

The original contributions presented in the study are included in the article/Supplementary Material, further inquiries can be directed to the corresponding authors.
