# Supplementary material for: Hyaluronic acid-modified liposomes Potentiated in-vivo anti-hepatocellular carcinoma of icaritin
Source: Front Pharmacol. 2024 Jul 11;15:1437515. doi: 10.3389/fphar.2024.1437515 (PMC11270019; doi:10.3389/fphar.2024.1437515)
Supplement: Supplementary file 1 [file DataSheet1.docx]

Supplementary Material

# Hyaluronic Acid-modified Liposomes Potentiated In-vivo Anti-hepatocellular Carcinoma of Icaritin

Xiaoduan Sun^1, 2, 3, †^, Zhenzhen He^3, †^, Ruilin Lu^4, †^, Zhongbing Liu^3^, Sawitree Chiampanichayakul^1, 5, 6^, Songyot Anuchapreeda^1, 5, 6^, Jun Jiang^7^, Singkome Tima^1, 5, 6, *^, Zhirong Zhong^3, 8, *^

^1^Department of Medical Technology, Faculty of Associated Medical Sciences, Chiang Mai University, Chiang Mai, Thailand.

^2^Department of Pharmacy, The Affiliated Hospital of Southwest Medical University, Luzhou, Sichuan, China.

^3^Key Laboratory of Medical Electrophysiology, Ministry of Education, School of Pharmacy, Southwest Medical University, Luzhou, Sichuan, China.

^4^Suining First People's Hospital, Suining, Sichuan, China.

^5^Cancer Research Unit of Associated Medical Sciences (AMS-CRU), Chiang Mai University, Chiang Mai, Thailand.

^6^Center of Excellence in Pharmaceutical Nanotechnology, Faculty of Pharmacy, Chiang Mai University, Chiang Mai, Thailand.

^7^Department of General Surgery (Thyroid Surgery), The Affiliated Hospital of Southwest Medical University, Luzhou, Sichuan, China.

^8^Central Nervous System Drug Key Laboratory of Sichuan Province, Luzhou, Sichuan, China.

^†^These authors have contributed equally to this work.

*** Correspondence:**Singkome Tima
[singkome.tima@cmu.ac.th](mailto:singkome.tima@cmu.ac.th)

Zhirong Zhong

[zhongzhirong@126.com](mailto:zhongzhirong@126.com)

**1. Supplementary methods**

**1.1 Synthesis of HA-Chol**

**Figure. S1** Synthetic route of HA-Chol copolymer.

Cholesterol (compound 1, 3.866 g, 10 mmol) was dissolved in 80 mL of anhydrous dichloromethane (DCM) at 0 °C. Then 2.25 mL of triethylamine (15 mmol) and p-toluenesulfonyl chloride (2.86 g, 15 mmol) were slowly added to the solution of 4-dimethylaminopyridine (DMAP, 120 mg, 1 mmol) as the catalyst at 0 °C. This compound was stirred at room temperature for overnight and washed two times orderly with HCl solution (1 N, 80 mL) and saturated NaCl solution (80 mL). The organic phase was then dried with anhydrous Na_2_SO_4_ and evaporated in vacuum. The crude product was further purified by the silica column chromatography (petroleum ether/ethyl acetate 4:1).

The preformed compound 2 (4.32 g, 84 mmol) was dissolved in anhydrous 1,4-dioxane (630 mL) and mixed with 30 mL of tri(ethyleneglycol) (222 mmol). The reaction kept overnight under an argon atmosphere and the thin-layer chromatography was used to confirm the endpoint of reaction. After removing 1,4-dioxane by evaporation, the left was dissolved into 100 mL chloroform and extracted two times with saturated NaHCO_3_ aqueous solution (100 mL), then washed two times with saturated NaCl solution (100 mL). The organic layer was dried with anhydrous Na_2_SO_4_. Chloroform was evaporated by rotary evaporation under vacuum condition. Then the crude product was purified with silica gel chromatography (petroleum ether/ethyl acetate 1:1).

HA (1 g), N,N’-dicyclohexylcarbodiimide (DCC, 0.5 g), and 4-dimethylaminopyridine (DMAP, 0.3 g) were dissolved in distilled water: N,N-dimethylformamide (1:3, v/v) and stirred for 2.5 hours to activate the carboxyl group of HA. After activation, the previous compound 3 (0.8 g) dissolved in N,N-dimethylformamide (80 mL) was added drop wise to the activated HA solution. After stirring for 48 hours, the solution was dialyzed against distilled water: tetrahydrofuran (1:1, v/v) solution and water, respectively. HA–Chol conjugates were obtained follow­ing freeze-drying.

## 1.2 Expression level of CD44

After incubated for 24 h, HepG2, Huh7 and L02 cells were used to check their CD44 expression levels by Western blot assay and Flow cytometry. In detail, RIPA buffer was used to prepare the whole cell lysates. Protein quantification was conducted through the BCA protein assay. Samples containing equal protein amounts (30 µg) from the lysates were subjected to separation via SDS-PAGE and then transferred onto nitrocellulose membranes. Following a 5% non-fat milk block, the blots were incubated with their primary antibodies against glyceraldehyde 3-phosphate dehydrogenase (GAPDH), p21, caspase 3a, p53 and CD44 overnight at 4 °C, and subsequently with peroxidase-conjugated secondary antibodies. The membranes were stripped and immunoblot bands were visualized through enhanced chemiluminescence (Millipore). GAPDH was used as a control for protein loading.

These 3 cells were stained with fluorochrome-conjugated monoclonal antibody CD44-FITC (Elabscience, E-AB-F1215C). The samples were incubated for 30 min in the dark and then washed with PBS for 2 times. All samples were resuspended in 500 μL PBS and analyzed by flow cytometer (Beckman, CytoFLEX LX) and FlowJo software (Version 10.8.1, Tree Star, San Carlos, CA).

**2. Supplementary results**

**2.1 Characterization of HA-Chol conjugate**

**2.1.1 *Synthesis of compound 2: (3S,8S,9S,10R,13R,14S,17R)-10,13-dimethyl-17-((R)-6-methylheptan-2-yl)-2,3,4,7,8,9,10,11,12,13,14,15,16,17-tetradecahydro-1H-cyclopenta[a]phenanthren-3-yl 4-methylbenzenesulfonate***

Yield: 50%. The NMR spectra were recorded on a Brooker 400 NMR Spectrometer. ^1^H NMR (400 MHz, CDCl_3_) δ 7.80 (d, *J* = 7.2 Hz, 2H), 7.33 (d, *J* = 7.6 Hz, 2H), 5.30 (s, 1H), 4.32 (s, 1H), 2.45 (s, 4H), 2.27 (d, *J* = 12.8 Hz, 1H), 1.97 (d, *J* = 16.6 Hz, 2H), 1.81 (d, *J* = 11.6 Hz, 3H), 1.73 (d, *J* = 11.2 Hz, 1H), 1.68–1.01 (m, 20H), 0.96 (s, 3H), 0.91 (s, 3H), 0.87 (s, 6H), 0.65 (s, 3H); ^13^C NMR (101 MHz, CDCl_3_) δ 144.4, 140.5, 140.1, 129.8, 129.8, 127.7, 127.7, 123.6, 82.4, 56.7, 56.1, 49.9, 42.3, 39.7, 39.5, 38.9, 36.9, 36.4, 36.2, 35.8, 31.9, 31.8, 28.7, 28.2, 28.0, 24.3, 23.8, 22.8, 22.6, 21.7, 21.0, 19.2, 18.7, 11.9.


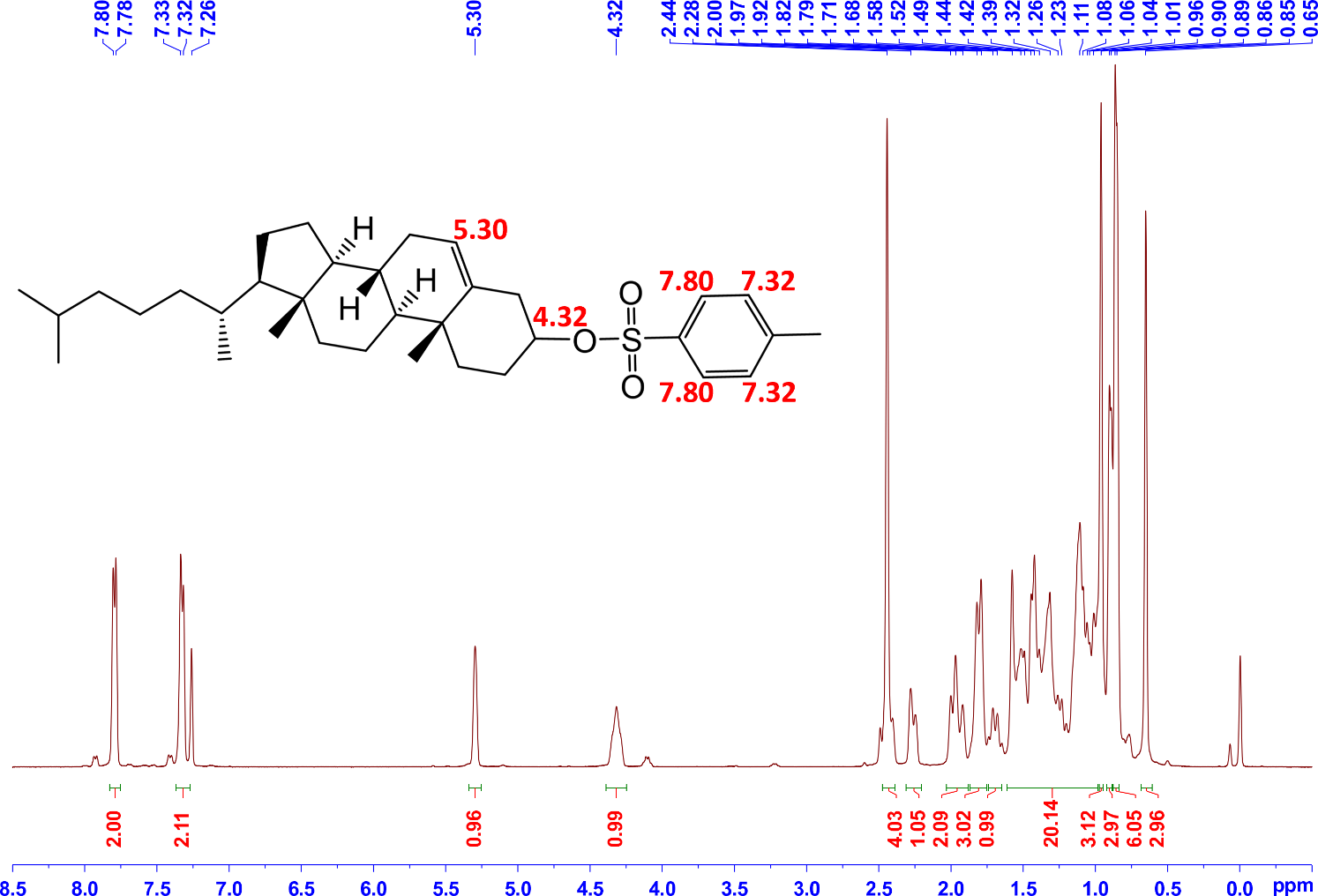


**Figure. S2** ^1^H NMR spectrum of synthetic compound 2

**2.1.2 *Synthesis of compound 3:* *2-(2-(2-(((3R,8S,9S,10R,13R,14S,17R)-10,13-dimethyl-17-((R)-6-methylheptan-2-yl)-2,3,4,7,8,9,10,11,12,13,14,15,16,17-tetradecahydro-1H-cyclopenta[a]phenanthren-3-yl)oxy)ethoxy)ethoxy)ethan-1-ol***

Yield: 57.4%. ^1^H NMR (400 MHz, CDCl_3_) δ 5.33 (s, 1H), 3.73–3.64 (m, 12H), 3.18 (s, 1H), 2.63 (s, 1H), 2.45–2.17 (m, 3H), 2.01–1.84 (m, 6H), 1.52–1.06 (m, 19H), 0.99 (s, 3H), 0.91 (s, 3H), 0.86 (s, 6H), 0.66 (s, 3H); ^13^C NMR (101 MHz, CDCl_3_) δ 140.4, 122.3, 80.3, 71.7, 70.8, 70.6, 70.3, 67.3, 61.3, 56.7, 56.5, 51.7, 41.7, 40.2, 39.9, 39.6, 37.7, 37.5, 36.1, 35.8, 32.0, 31.8, 29.5, 28.1, 26.3, 25.9, 24.6, 23.5, 23.2, 21.1, 19.6, 19.3, 12.0.


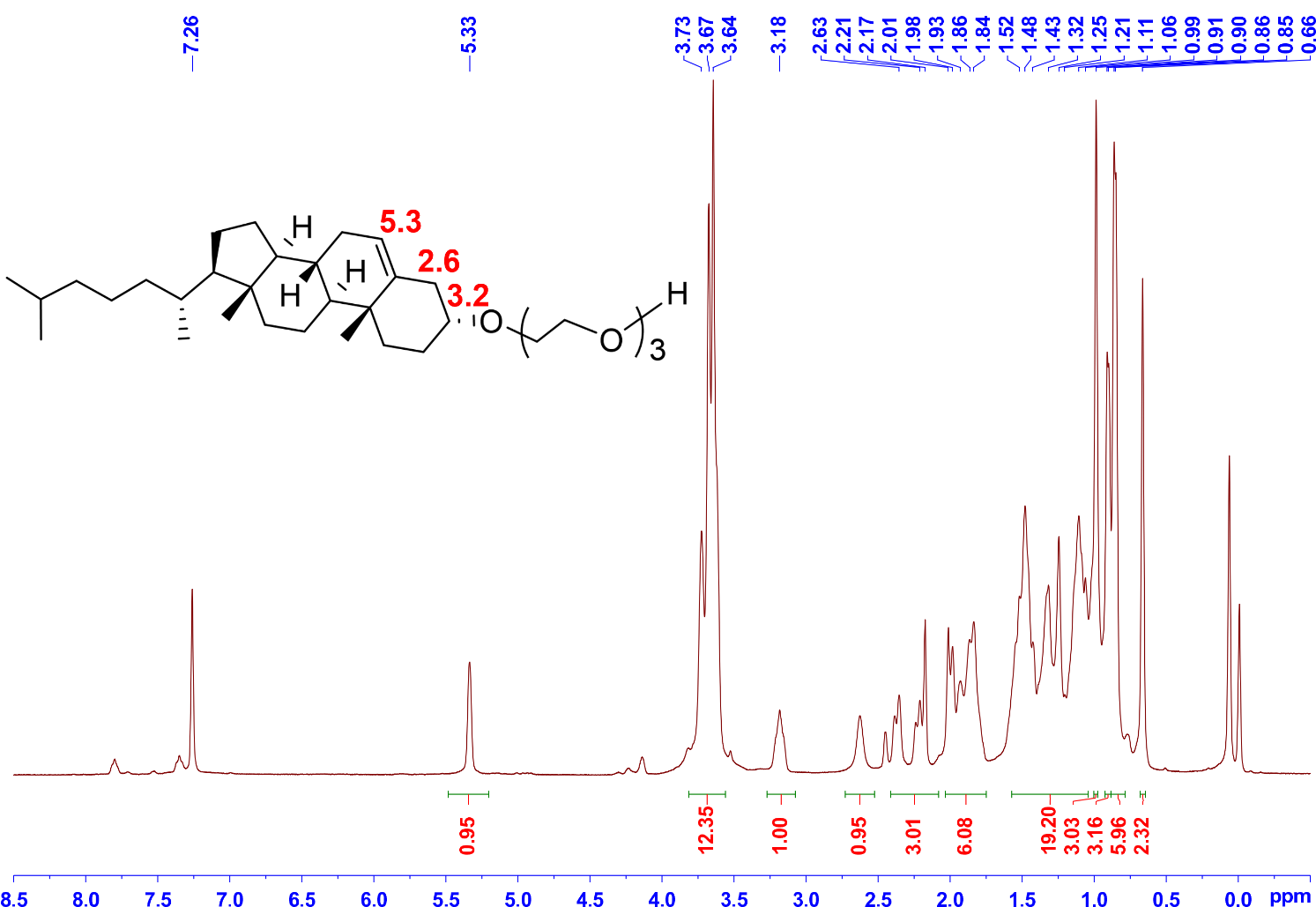


**Figure. S3** ^1^H NMR spectrum of synthetic compound 3

**2.1.3 *Synthesis of compound 4: (2S,4S,5R,6S)-6-(((2S,3R,4S,5S,6R)-3-acetamido-5-hydroxy-6-(hydroxymethyl)-2-methoxytetrahydro-2H-pyran-4-yl)oxy)-4,5-dihydroxy-3-methyltetrahydro-2H-pyran-2-carboxylic acid compound with 1-((2S,3S,4S,5R)-4,5,6-trihydroxy-3-methyltetrahydro-2H-pyran-2-yl)ethan-1-one and 2-(2-(2-(((8S,9S,10R,13R,14S,17R)-10,13-dimethyl-17-((R)-6-methylheptan-2-yl)-2,3,4,7,8,9,10,11,12,13,14,15,16,17-tetradecahydro-1H-cyclopenta[a]phenanthren-3-yl)oxy)ethoxy)ethoxy)ethan-1-ol (1:1:1)***


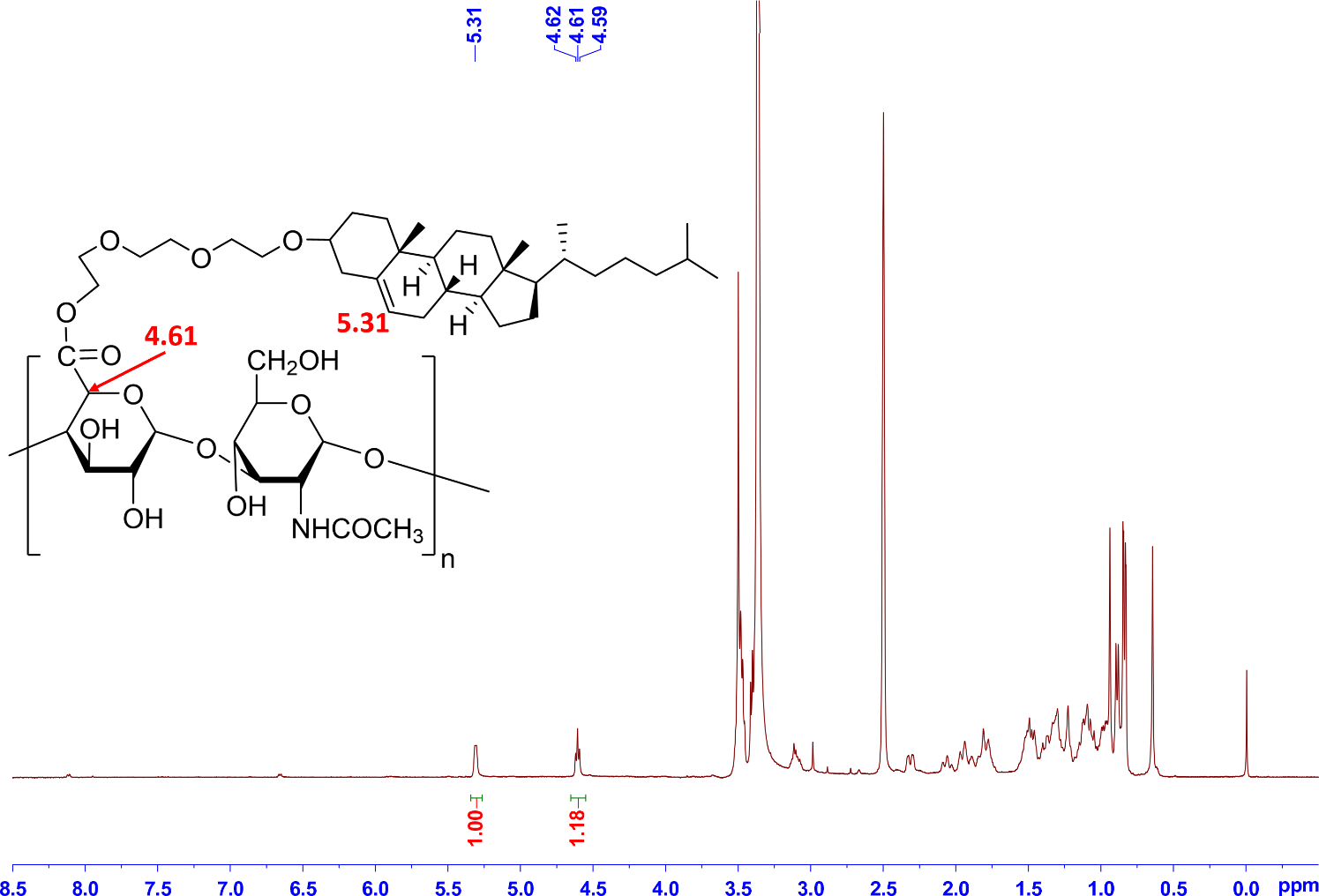


**Figure. S4** ^1^H NMR spectrum of synthetic compound 4

The methine group of Chol (δ=5.32 ppm [1H, –CH]) and the methine group in the sugar unit of HA (δ=4.62 ppm [1H, –COCH]) were confirmed in the ^1^H NMR spectrum of HA–Chol, indicating successful synthesis of HA–Chol.


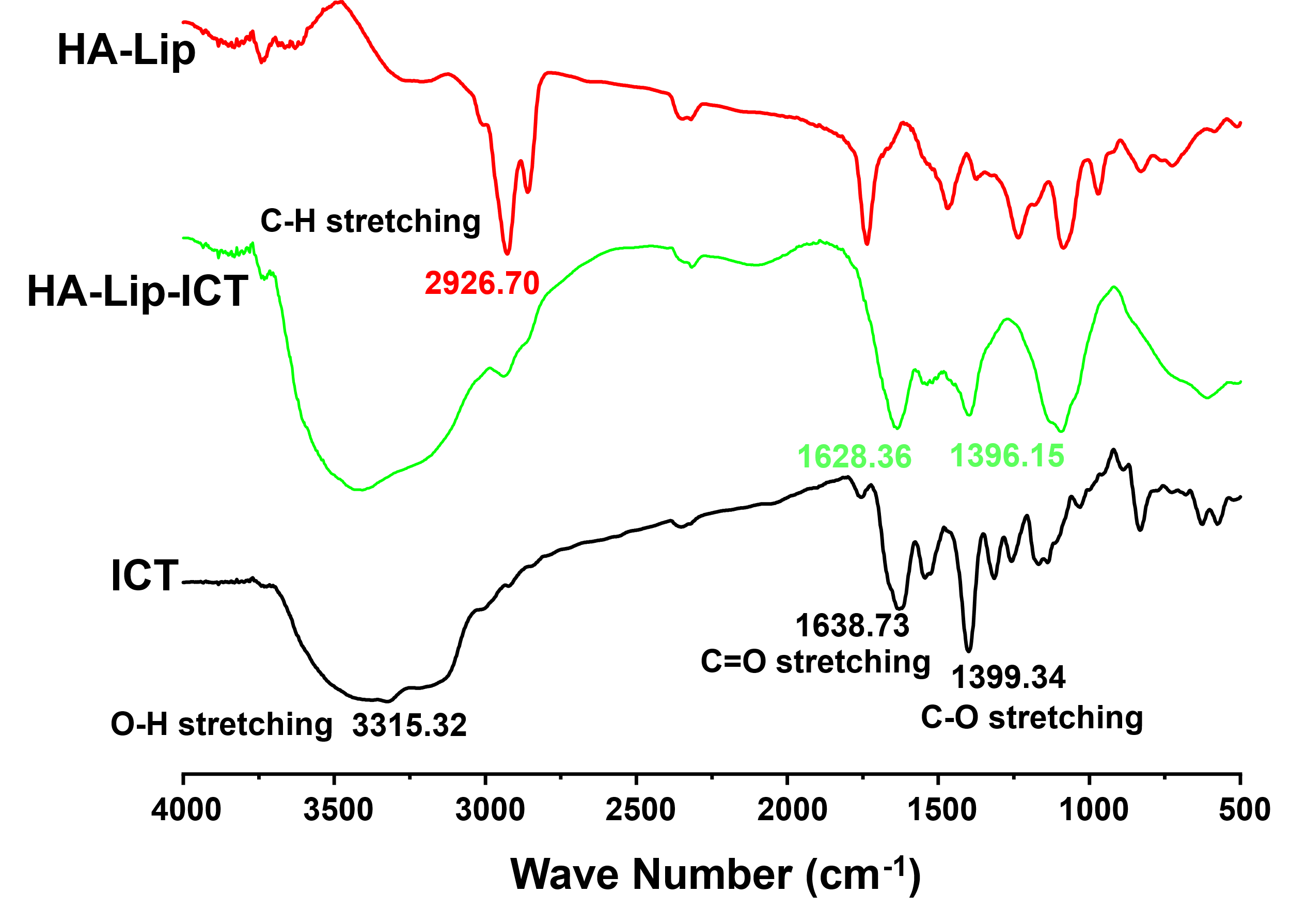


**Figure. S5** FTIR spectra of HA-Lip, HA-Lip-ICT and ICT.


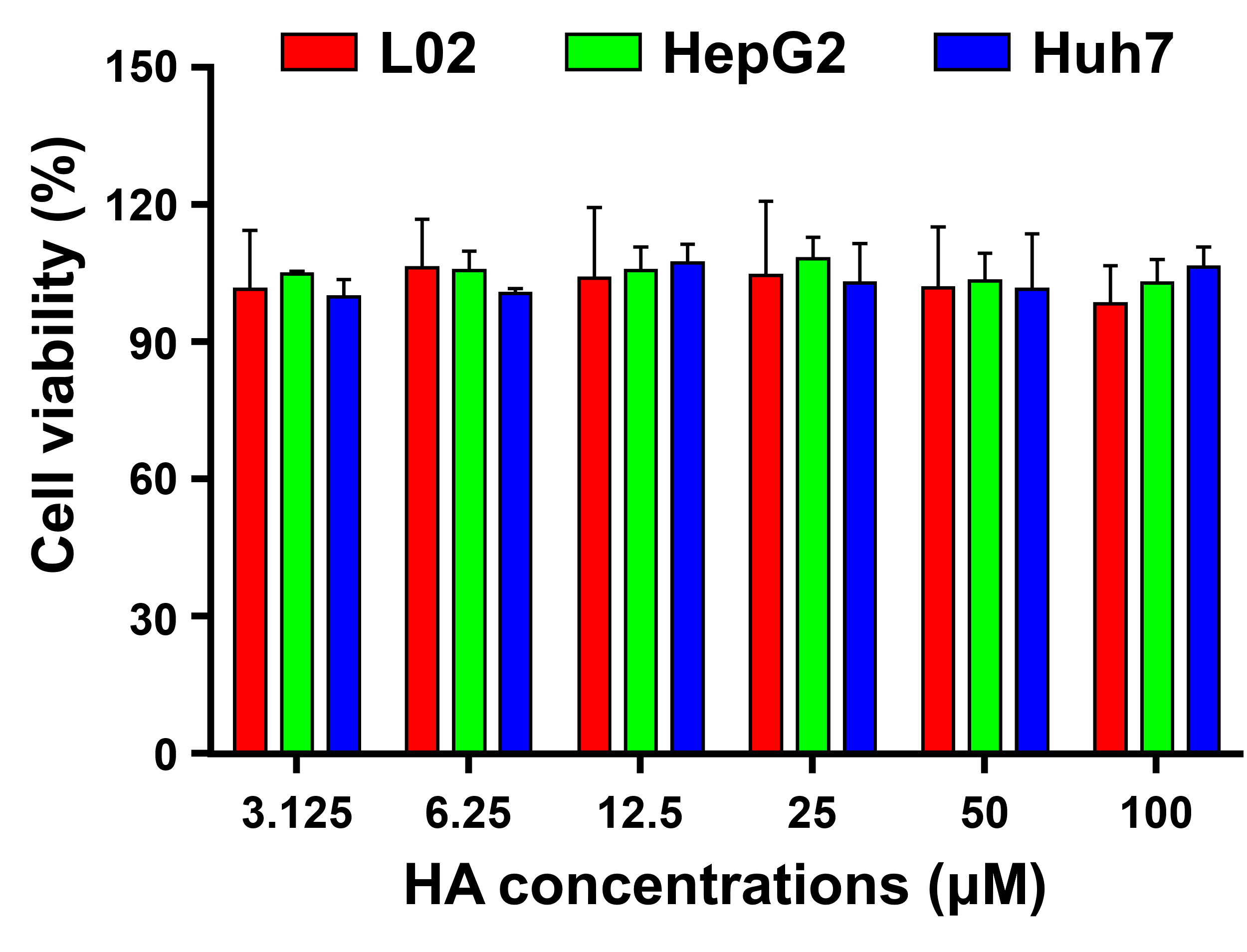


**Figure. S6** Cell viability of Huh7 cells, HepG2 cells and L02 cells after incubated with varied concentrations of HA-Chol for 48 h.


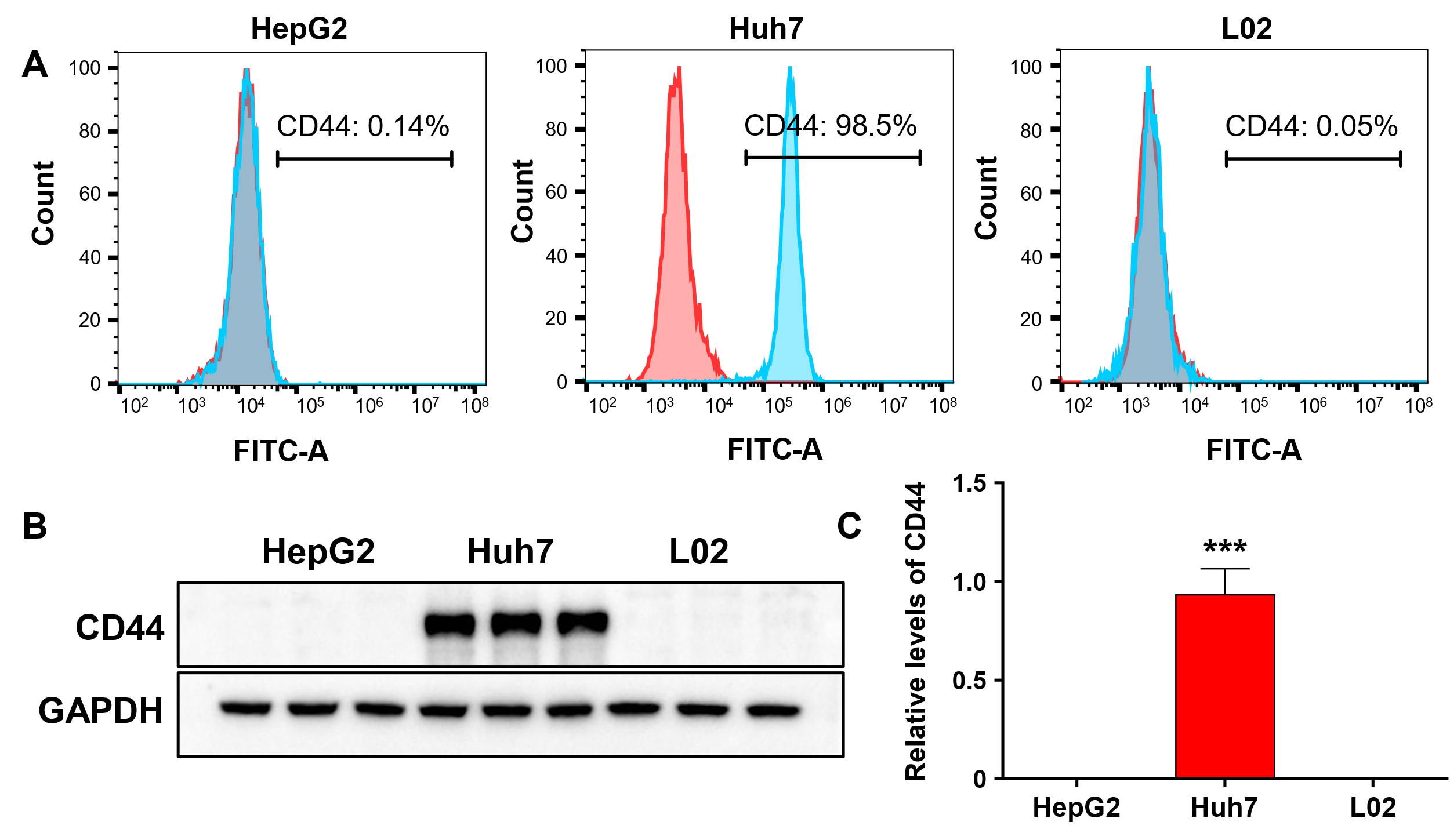


**Figure. S7** CD44 expression levels in HepG2 cells, Huh7 cells and L02 cells analyzed by flowcytometry (A) and western blot assay (B). Relative protein expression levels of CD44 (C) (n = 3). ****p* < 0.001.

**Table S1**. Stability of Empty Lip, Lip-ICT, and HA-Lip-ICT at 4 °C.

| Sample | Days | Particle size (nm) | Polydispersity index | EE (%) |
| --- | --- | --- | --- | --- |
| Empty Lip | 0 | 164.33 ± 1.89 | 0.147 ± 0.005 |  |
|  | 5 | 164.57 ± 1.79 | 0.145 ± 0.006 |  |
|  | 10 | 165.80 ± 2.91 | 0.147 ± 0.008 |  |
| Lip-ICT | 0 | 179.53 ± 1.83 | 0.121 ± 0.007 | 75.83 ± 1.08 |
|  | 5 | 171.33 ± 1.71 | 0.153 ± 0.005 | 75.78 ± 0.71 |
|  | 10 | 172.27 ± 2.84 | 0.135 ± 0.006 | 75.71 ± 0.60 |
| HA-Lip-ICT | 0 | 208.83 ± 2.43 | 0.165 ± 0.007 | 81.21 ± 1.06 |
|  | 5 | 209.47 ± 2.57 | 0.174 ± 0.008 | 81.91 ± 1.02 |
|  | 10 | 211.93 ± 2.73 | 0.187 ± 0.007 | 82.84 ± 0.75 |

Data are shown as mean ± SD. EE, encapsulation efficiency (%); HA, hyaluronic acid; Lip, liposomes; ICT, icaritin.

**Table S2**. Stability of Empty Lip, Lip-ICT, and HA-Lip-ICT at 25 °C.

| Sample | Days | Particle size (nm) | Polydispersity index | EE (%) |
| --- | --- | --- | --- | --- |
| Empty Lip | 0 | 174.33 ± 1.95 | 0.157 ± 0.007 |  |
|  | 5 | 334.77 ± 3.11 | 0.473 ± 0.017 |  |
|  | 10 |  |  |  |
| Lip-ICT | 0 | 219.53 ± 1.90 | 0.172 ± 0.009 | 75.83 ± 1.08 |
|  | 5 | 414.50 ± 3.44 | 0.586 ± 0.051 | 62.80 ± 0.84 |
|  | 10 |  |  |  |
| HA-Lip-ICT | 0 | 238.83 ± 2.43 | 0.194 ± 0.006 | 82.21 ± 1.06 |
|  | 5 | 451.53 ± 1.95 | 0.737 ± 0.059 | 71.52 ± 0.71 |
|  | 10 |  |  |  |

Data are shown as mean ± SD. EE, encapsulation efficiency (%); HA, hyaluronic acid; Lip, liposomes; ICT, icaritin.
